# *Yersinia enterocolitica* Targets Cells of the Innate and Adaptive Immune System by Injection of Yops in a Mouse Infection Model

**DOI:** 10.1371/journal.ppat.1000551

**Published:** 2009-08-14

**Authors:** Martin Köberle, Annegret Klein-Günther, Monika Schütz, Michaela Fritz, Susanne Berchtold, Eva Tolosa, Ingo B. Autenrieth, Erwin Bohn

**Affiliations:** 1 Institut für Medizinische Mikrobiologie und Hygiene, Universitätsklinikum Tübingen, Tübingen, Germany; 2 Hertie-Institut für klinische Hirnforschung, Universitätsklinikum Tübingen, Tübingen, Germany; 3 Zentrum für Molekulare Neurobiologie, Universität Hamburg, Hamburg, Germany; Tufts University School of Medicine, United States of America

## Abstract

*Yersinia enterocolitica* (Ye) evades the immune system of the host by injection of *Yersinia* outer proteins (Yops) via a type three secretion system into host cells. In this study, a reporter system comprising a YopE-β-lactamase hybrid protein and a fluorescent staining sensitive to β-lactamase cleavage was used to track Yop injection in cell culture and in an experimental Ye mouse infection model. Experiments with GD25, GD25-β1A, and HeLa cells demonstrated that β1-integrins and RhoGTPases play a role for Yop injection. As demonstrated by infection of splenocyte suspensions in vitro, injection of Yops appears to occur randomly into all types of leukocytes. In contrast, upon infection of mice, Yop injection was detected in 13% of F4/80^+^, 11% of CD11c^+^, 7% of CD49b^+^, 5% of Gr1^+^ cells, 2.3% of CD19^+^, and 2.6% of CD3^+^ cells. Taking the different abundance of these cell types in the spleen into account, the highest total number of Yop-injected cells represents B cells, particularly CD19^+^CD21^+^CD23^+^ follicular B cells, followed by neutrophils, dendritic cells, and macrophages, suggesting a distinct cellular tropism of Ye. Yop-injected B cells displayed a significantly increased expression of CD69 compared to non-Yop-injected B cells, indicating activation of these cells by Ye. Infection of IFN-γR (receptor)- and TNFRp55-deficient mice resulted in increased numbers of Yop-injected spleen cells for yet unknown reasons. The YopE-β-lactamase hybrid protein reporter system provides new insights into the modulation of host cell and immune responses by Ye Yops.

## Author Summary

An important strategy of *Yersinia enterocolitica* (Ye) to suppress the immune defense is to inject bacterial proteins (*Yersinia* outer proteins, Yops) after cell contact directly into host cells, which affects their functions. However, tracking of cells in which Yop injection occurred has only been described for *Yersinia pestis* thus far. We adapted the described reporter system specifically for the use of infections with Ye and report the usefulness and limitations of this system. Using cell culture experiments, we demonstrated that β1-integrins and the RhoGTPases RhoA and Rac1 are involved in Yop injection. Since cell culture experiments also revealed that Yop injection is detectable in a similar manner into all subpopulations of the spleen, the system can be used to detect interaction of bacteria with host cells in vivo. In a mouse infection model we found that follicular B cells, granulocytes, macrophages, and dendritic cells are the main targets of Yop injection. Interestingly, Yop-injected B cells displayed an increased activation as indicated by increased CD69 expression. In contrast, interaction of bacteria with T cells seems to be rather a rare event. In immunocompromised gene-targeted mice we found increased frequencies of Yop-injected host cells for yet unknown reasons. Taken together, this novel reporter system represents a powerful tool to further study interaction of host cells with Ye.

## Introduction

*Yersinia enterocolitica* (Ye) is an enteropathogenic bacterium that causes gastrointestinal disorders such as enteritis and enterocolitis as well as extraintestinal manifestations such as lymphadenitis, reactive arthritis, and septicemia [1],[2]. Ye has been demonstrated to multiply extracellularly in host tissue. To accomplish this, Ye needs to evade the host's immune defense. Beside other virulence factors, Ye evolved a type III secretion system (TTSS) consisting of an injectisome and effector proteins the latter of which are injected into host cells [3]. The injection of effectors into host cells via a TTSS injectisome is a common strategy of pathogenic bacteria to counteract the host's immune response [4]. The TTSS injectisome is complex ATP-driven protein-export machinery. Built of ring shaped proteins, the basal body is providing a channel through the bacterial membranes and the periplasm or the peptidoglycan wall, respectively. The injectisome is terminating in a needle-like structure that is protruding from the bacterial surface [5],[6]. Thus, pore-forming proteins enable the injection of the effector proteins through the membrane of host target cells [7],[8].

The TTSS is crucial for *Yersinia* virulence [9]. Ye injects at least six effector Yops into host cells. YopP/J is a potent inhibitor of the NF-κB and the MAPK signaling pathways and thus inhibits downstream effects of these pathways such as proinflammatory responses or antigen uptake [10]–[16]. In addition YopP induces apoptosis in macrophages and dendritic cells [17]–[20]. YopE, YopT and YopO affect RhoGTPase functions which leads to actin cytoskeleton disruption and together with YopH, a tyrosine phosphatase which targets different eukaryotic kinases, promote inhibition of phagocytosis [21]. In addition, phosphatase activity of YopH counteracts T cell activation [22],[23]. YopM is known to interact directly with protein kinase C-like 2 (PRK2) and ribosomal S6 protein kinase 1 (RSK1), the function of YopM is so far elusive [24].

Despite well defined and profound in vitro effects of Yops, the contribution of some Yops to establish successful infection in a mouse infection model seems to be rather insignificant (YopP/O/T), while others (YopE/H/M) are of great importance for virulence of Ye [9]. This highlights the necessity to understand *Yersinia* pathogenicity in the context of whole organism infection. It also creates the need for a tool to display the sites of interaction between host organism and pathogen.

Various approaches have been taken to enable monitoring of type three secretion into eukaryotic cells by *Yersinia* and other pathogens during infection in cell culture. TTSS effectors have been detected in host cells by immunocytochemistry and western blot [8],[25] and fusions of effectors with GFP [26], the adenylate cyclase (CyA) [27], Elk-tag [28] or Cre-recombinase [29]. Thus, yersiniae expressing YopE-GFP could be used to detect fluorescent bacteria in cell culture and mouse infection, but clear Yop injection into host cells could not be shown or quantified [30]. The YopE-CyA fusion was useful to detect Yop injection directly in vitro and was utilized to demonstrate the requirement of YopB and YopD for Yop injection by quantification of cAMP levels in infected cells [27]. Briones et al. used a SopE-Cre reporter system which allowed the visualization of Salmonella Sop injection at least in a low number of cultured cells [29]. All these systems have enabled interesting discoveries, but failed to detect Yop injection on a single cell level and are not suitable for the quantitative detection of targeted cells in mouse infection models.

Recently, a new reporter system for monitoring type III secretion of bacterial proteins into host cells has been described to study effector injection of enteric *Escherichia coli* [31] and *Salmonella enterica* [32] as well as *Yersinia pestis* [30]. The reporter systems consisted of translational fusions of a whole or truncated TTSS effector protein with mature *E. coli* TEM-1 β-lactamase. Infected cells were stained in these studies with the lipophilic CCF2-AM [33], an esterified form of the CCF2 substrate. After entry into the cells endogenous cytoplasmic esterases rapidly convert CCF2-AM into its negatively charged form CCF2, which is retained in the cytosol. CCF2 is a fluorescence resonance energy transfer (FRET) substrate, which consists of a cephalosporin core linking 7-hydroxycoumarin to fluorescein. Excitation of the coumarin moiety at 409 nm results in a FRET to the fluorescein residue leading to emission of light with a wavelength of 520 nm (green fluorescence). Cleavage of the CCF2 substrate by TEM-1 β-lactamase separates the two fluorescent moieties and interrupts the FRET between them. Excitation of the coumarin residue now leads to the emission of light at a wavelength of 447 nm (blue fluorescence). Marketon et al. [30] used an expression vector which expressed YopM-β-lactamase fusion protein in *Y. pestis* for infection experiments of mice. Using this system it was shown that Yops were predominantly injected into granulocytes, dendritic cells and macrophages while injection into T and B cells was found to be a rare event. Geddes et al. [32] used translational fusions of bla and effectors of the SPI-1/2 pathogenicity islands as reporters and demonstrated that *Salmonella* targets preferentially granulocytes in the spleen after mouse infection.

To investigate into which immune cells Yops are injected during *Y. enterocolitica* infection in vivo, a Bla reporter system was established to track Yop injection in infection in cell culture and in an experimental mouse infection model. The established system was validated as a powerful tool to investigate Yop injection in cell culture as well as in a mouse infection model.

## Results

### Construction of a reporter system for tracking *Y. enterocolitica* Yop injection into host cells

**Figure 1:**
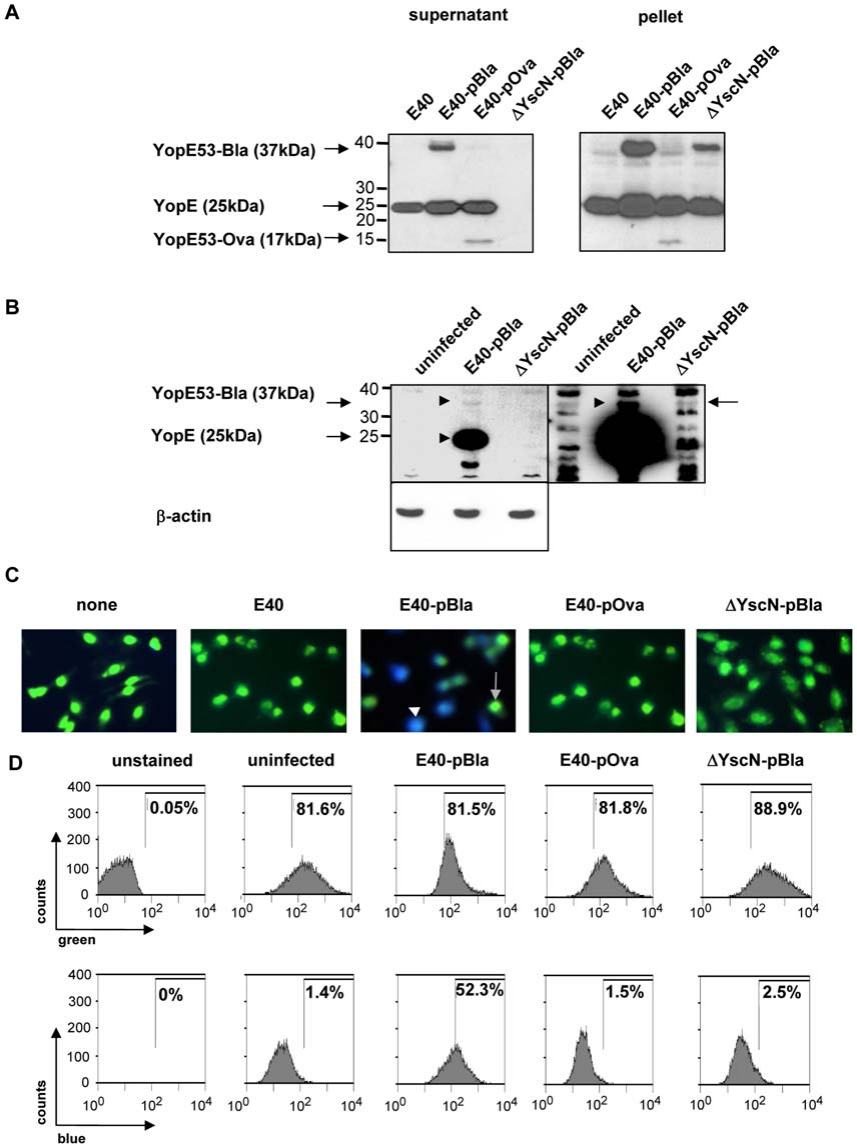
Establishment of a β-lactamase reporter system and detection of Yop injection in HeLa cells. (A) Secreted (left panel) and intracellular (right panel) YopE, YopE53-Bla and YopE53-Ova hybrid proteins derived from supernatants or pellets, respectively, of bacterial cultures were detected by immunoblot using anti-YopE antibodies. To determine Yop injection into HeLa cells, cells were infected for 1 h (MOI 100) and injection of YopE hybrid proteins was detected (B) by immunoblot using anti-YopE and anti-actin antibodies. To visualize YopE53-Bla exposure time was extended (right panel). (C) Yop injection was detected as blue fluorescence by staining of cells with green fluorescent TEM-1 β-lactamase sensitive FRET substrate CCF4-AM and subsequent visualization by immunofluorescence microscopy. Blue and green fluorescence recordings were overlaid to get composite pictures. Blue and green cells are indicated with white and grey arrowheads, respectively. Alternatively, fluorescence of cells was detected by (D) flow cytometry. After flow cytometry data were analyzed and depicted as histograms. The upper panel shows uptake of CCF4-AM (green) by cells. The horizontal bar indicates the gating used to determine the number of β-lactamase positive cells revealing Yop-injection (blue) in the lower panel. Representative data of one of four experiments are shown. For note: percentages of blue or green fluorescent cells found in the histograms as depicted in this or other figures represent the results of this single experiment and are therefore discrepant to the means summarizing always several experiments as described in the results section.

To establish a reporter system to detect Yop injection by Ye, the expression vector pMK-Bla was transformed into E40 *Δasd*, resulting in the strain E40-pBla, secreting a YopE53-β-lactamase fusion protein (see Materials and Methods as well as Table 1). As a control, a strain also harboring pMK-Bla but deficient in Yop secretion (ΔYscN-pBla) was employed. As a further control the plasmid pMK-Ova, which encodes for a translational fusion of YopE53 with the ovalbumin aa 247–355 fragment, was transformed into E40 *Δasd* resulting in E40-pOva. Expression and secretion of YopE and the fusion proteins YopE53-Bla and YopE53-Ova by the strains E40-pBla, E40-pOva and ΔYscN-pBla was analyzed by immunoblotting. The data depicted in Figure 1A indicate that YopE wild type and hybrid proteins were expressed and secreted into the supernatant except by the ΔYscN-pBla mutant which is deficient for Yop secretion. To confirm injection of YopE and YopE53-Bla, HeLa cells were mock-infected or infected with E40-pBla or ΔYscN-pBla and injection assays were performed to detect YopE and YopE53-Bla by immunoblot using anti-YopE antibodies. YopE injection was detectable after infection with E40-pBla but not after infection with ΔYscN-pBla (Figure 1B left panel). In contrast, a YopE-Bla band was hardly visible. Only long overexposure led to a clearly visible YopE53-Bla band in E40-pBla, but not ΔYscN-pBla infected cells (Figure 1B right panel). This shows that YopE53-Bla is translocated into cells but with a much lower efficacy than the wildtype YopE.

**Table 1:** Plasmids and bacterial strains used in this study.

Designation	Genotype or Description	Reference or Source
*Plasmids*		
pBME53-YopT	*pACYC184* derivative containing *Hind*III-*sycE*-*yopE*_53_-*BamH*I-*yopT*-*Sal*I. Cm^R^	[56]
pBME53-Cre	Derivative of pBME53-YopT. *Bam*HI-*yopT* – *Sal*I fragment was replaced by *Bam*HI –*nls-cre*–*Sal*I resulting in *Hind*III- *sycE*-*yopE*_53_-*BamH*I-*nls-cre*-*Sal*I. Cm^R^	This study
pIV2	Cloning vector based on a small kryptic plasmid from *Y. enterocolitica* biogroup 1A strain 29807 modified with a kanamycin resistance marker and a pBluescript MCS. Kan^R^	[57]
pIV2-SycE-YopE53-Cre	Derivative of pIV2 with insertion of *Hind*III *sycE*-*yopE*_53_-*BamH*I-*nls-cre*-*Sal*I into MCS. Kan^R^	This study
pMK4	Derivative of pIV2-SycE-YopE53-Cre with insertion of *asd* under control of its own promoter between the *Apa*I and *Xho*I sites. Kan^R^	This study
pMK1	*Sal*I*-asd* 3′UTR-*Eco*RI-*asd* 5′UTR-*Xba*I in pBluescript II SK(+). Amp^R^	This study
pMK2	*Sal*I*-asd* 3′UTR-*Eco*RI-*asd* 5′UTR-*Xba*I in pMRS101. Cm^R^	This study
pMK3	*asd* mutator plasmid, derived from pMK2 by *Not*I digestion and relegation. Cm^R^	This study
pMRS101	Mutator plasmid. Cm^R^	[59]
pBME53-Bla	Derivative of pBME53-YopT. *Bam*HI-*yopT* – *Sal*I fragment was replaced by *Bam*HI–*bla*– *Sal*I resulting in *Hind*III sycE-*yopE*_53_-*BamH*I-*bla*-*Sal*I. Cm^R^	This study
pMK-Bla	*Hind*III sycE-*yopE*_53_-*Bam*HI-*NLS-Cre-Sal*I fragment of pMK4 was replaced by a *Hind*III sycE-*yopE*_53_-*Bam*HI-*bla*-*Sal*I fragment of pBM53-Bla. Kan^R^	This study
pYopE_1–138_Ova_247–355_	*pACYC184* derivative containing *Hind*III- *sycE*-*yopE*_138_-*Bam*HI-*ova*_147–355_- *Sal*I	[58]
pBME53-Ova	Derivative of pBME53-YopT. *Bam*HI-*yopT* – *Sal*I fragment was replaced by *Bam*HI– *ova*_147–355_- *Sal*I. Cm^R^	This study
pMK-Ova	Derivative of pMK-Bla. *Bam*HI-*bla*-*Sal*I was replaced by *Bam*HI– *ova*_147–355_- *Sal*I from pBME53-Ova. Kan^R^	This study
pMSL41	pYV40 with deletion in the *yscN* gene (*yscNΔ169–177*). Ars^R^	[27],[59]
*Y. enterocolitica* E40 strains		
E40	Serotype O:9 patient isolate. Nal^R^, Ars^R^	[27]
E40 Δ*asd*	E40 strain with *asd* gene knockout, deficient in L-aspartate-dehydrogenase expression. Nal^R^, Ars^R^, DAP^aux^	This study
E40-pBla	E40 Δ*asd* strain transformed by pMK-Bla. Nal^R^, Ars^R^, Kan^R^	This study
pYV^−^ *Δasd* pBla	E40 Δ*asd* pMK-Bla strain without pYV40 virulence plasmid. Nal^R^, Kan^R^	This study
ΔYscN-pBla	pYV^−^ *Δasd* pBla strain transformed by pMSL41. Nal^R^, Ars^R^, Kan^R^	This study
E40-pOva	E40 Δ*asd* strain transformed by pMK-Ova. Nal^R^, Ars^R^, Kan^R^	This study

### Detection of Yop injection into epithelial cells in vitro

To investigate the usability of the system to assay Yop injection into host cells, HeLa cells were infected with the E40-pBla, E40-pOva or ΔYscN-pBla mutant strains and subsequently stained with CCF4-AM. In the presented study CCF4-AM was used instead of CCF2-AM that was utilized in former studies, because according to the manufacturer the FRET is slightly stronger with this dye, resulting in less background staining. Microscopic analysis of the uninfected cells as well as the controls including infection with E40-pOva or ΔYscN-pBla showed only green net fluorescence resulting from intact FRET within CCF4 (Figure 1C). In contrast, after E40-pBla infection, approximately half of the cells showed a pronounced blue net fluorescence resulting from FRET disruption by β-lactamase, suggesting that injection of the YopE53-Bla hybrid protein occurred (Figure 1C).

To quantify cell numbers and fluorescence signal intensity, flow cytometry was used. Green fluorescence resulting from CCF4-AM dye substrate uptake, de-esterification and retention in infected HeLa cells was equal to that observed in uninfected cells (Figure 1D). It also reflects the viability of cells because dead cells cannot retain the CCF4-dye (data not shown). After infection of HeLa cells for 1 hour with E40-pBla (MOI 50), blue fluorescence resulting from the cleavage of CCF4 was detectable in 58.9±11% of the viable cells. In contrast, infection of HeLa cells with E40-pOva or ΔYscN-pBla resulted in 0.48±0.3% or 2.5±2.4% blue cells, indicating that spontaneous cleavage of the substrate CCF4 after infection does not significantly occur and that Yop secretion is essential to detect Yop injection into host cells.

### Role of β1-integrins for Yop injection

**Figure 2:**
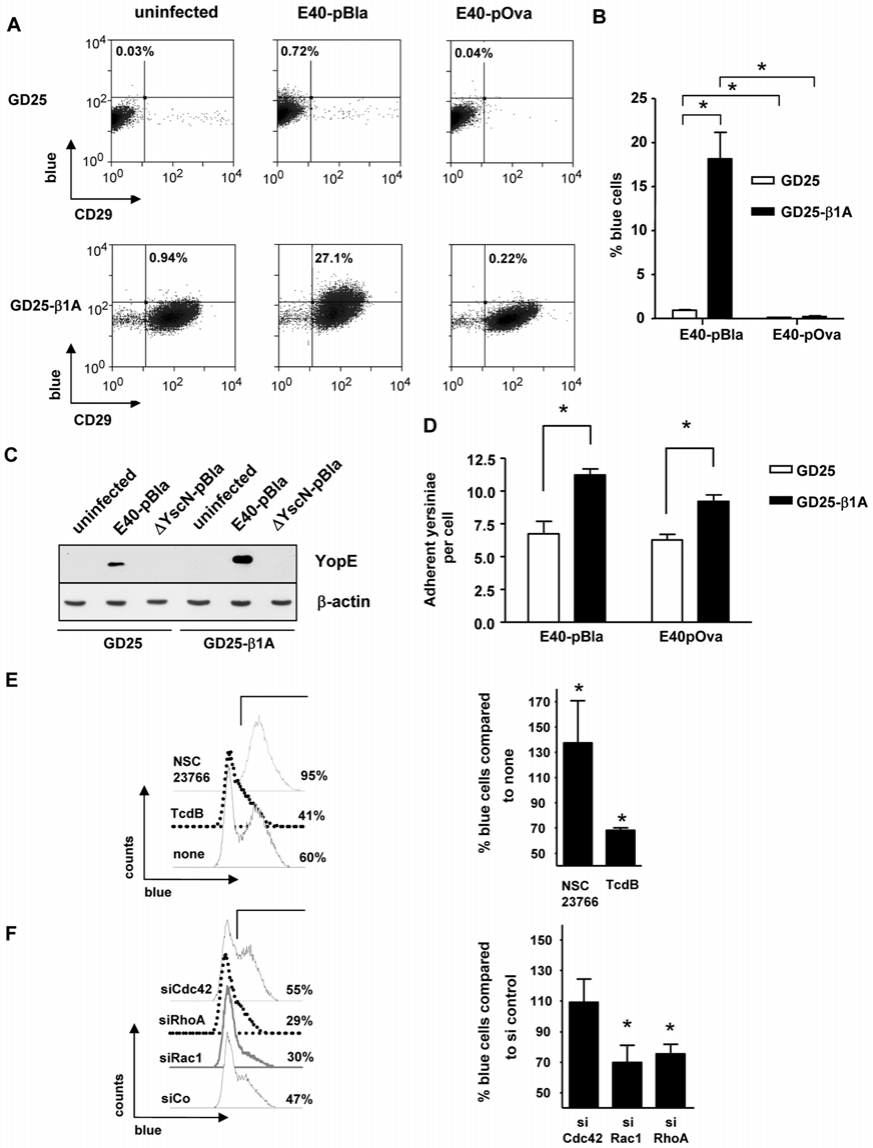
Role of β1-integrins for Yop injection. GD25 and GD25-β1A cells were infected with E40-pOva or E40-pBla for one hour and subsequently stained with CCF4-AM and analyzed by flow cytometry. Living cells with high green fluorescence of CCF substrate were gated and are depicted in (A) dot blots. Data show the expression of β1-integrins (CD29) and blue fluorescence intensity of the coumarin product of uninfected or infected GD25 (upper panel) and GD25-β1A cells (lower panel) of one experiment. (B) Data show percentage of blue cells as the mean±SEM summarizing four independent experiments. Asterisks indicate significant differences (One-way ANOVA with Bonferroni corrections, p<0.001). (C) Injection of YopE into cells was detected by immunoblot using anti-YopE and anti-actin antibodies. (D) Adhesion assays were performed after infection of indicated cells with E40-pBla or E40-pOva for one hour. Bacteria and cells were stained with fuchsine and adherent bacteria per cell were counted. Data depict the mean and SD of two independent experiments. Hela cells were either (E) pretreated with TcdB (200 ng/ml) for 2 hours or pretreated with NSC23766 (100 µM) for three hours or (F) transfected with indicated siRNAs for 48 hours. Cells were subsequently infected with E40-pBla (MOI 50) for one hour and stained with CCF-AM and analyzed by flow cytometry. Data show percentage of blue cells as the mean±SEM of two (E) or three (F) independent experiments. Asterisks indicate significant differences compared to control (Paired t-test with control, p<0.05).

Adhesion of Ye to host cells is mediated by interaction of host cell β1-integrins with *Yersinia* invasin protein or YadA (indirectly via collagen); moreover, adhesion of *Ye* to host cells is a prerequisite for Yop injection into host cells [34]. To investigate whether β1-integrins are essential for Yop injection, infection experiments were performed with the fibroblast cell line GD25, lacking β1-integrins and the cell line GD25-β1A, overexpressing β1-integrins as indicated in Figure 2A. Infection of GD25 cells with E40-pBla or E40-pOva (MOI 50) for one hour resulted in 0.9±0.2% or 0.12±0.06% blue cells while infection of GD25-β1A cells yielded 18.2±6% or 0.22±0.12% blue cells, respectively (Figure 2B). The strong reduction (95%) of Yop injection into GD25 cells compared to GD25-β1A cells was also confirmed by detection of YopE by immunoblots after infection with E40-pBla (Figure 2C). To address whether reduced Yop injection into GD25 cells was due to reduced adhesion of *Ye* to GD25 compared to GD25-β1A cells, cell adhesion assays were performed. GD25 showed a modest (40%) but significant decrease of the number of adhering yersiniae compared to GD25-β1A cells (Figure 2D).

*C. difficile* toxin B (TcdB), an inhibitor of RhoA, was shown to inhibit Yop injection while the Rac inhibitor NSC23766 had no impact on Yop injection [35]. According to these data we hypothesized that RhoA might be crucial for Yop injection. To confirm the previous data, HeLa cells were pretreated with TcdB or NSC23766 and then infected with E40-pBla and stained with CCF4-AM (Figure 2E). Flow cytometry analysis revealed that the number of blue cells was significantly decreased by TcdB, but not by the Rac1 inhibitor NSC23766. Additionally, HeLa cells were transfected with control siRNA (siCo) or siRNAs specific for the RhoGTPases RhoA, Cdc42 or Rac1. The cells were subsequently infected with E40-pBla, stained with CCF4-AM and analyzed by flow cytometry (Figure 2E). Inhibition of RhoA and Rac1, but not Cdc42, significantly reduced the number of blue cells by 25–30% indicating that both Rac1 and RhoA play a role in Yop injection.

### Detection of Yop injection into leukocytes in vitro

**Figure 3:**
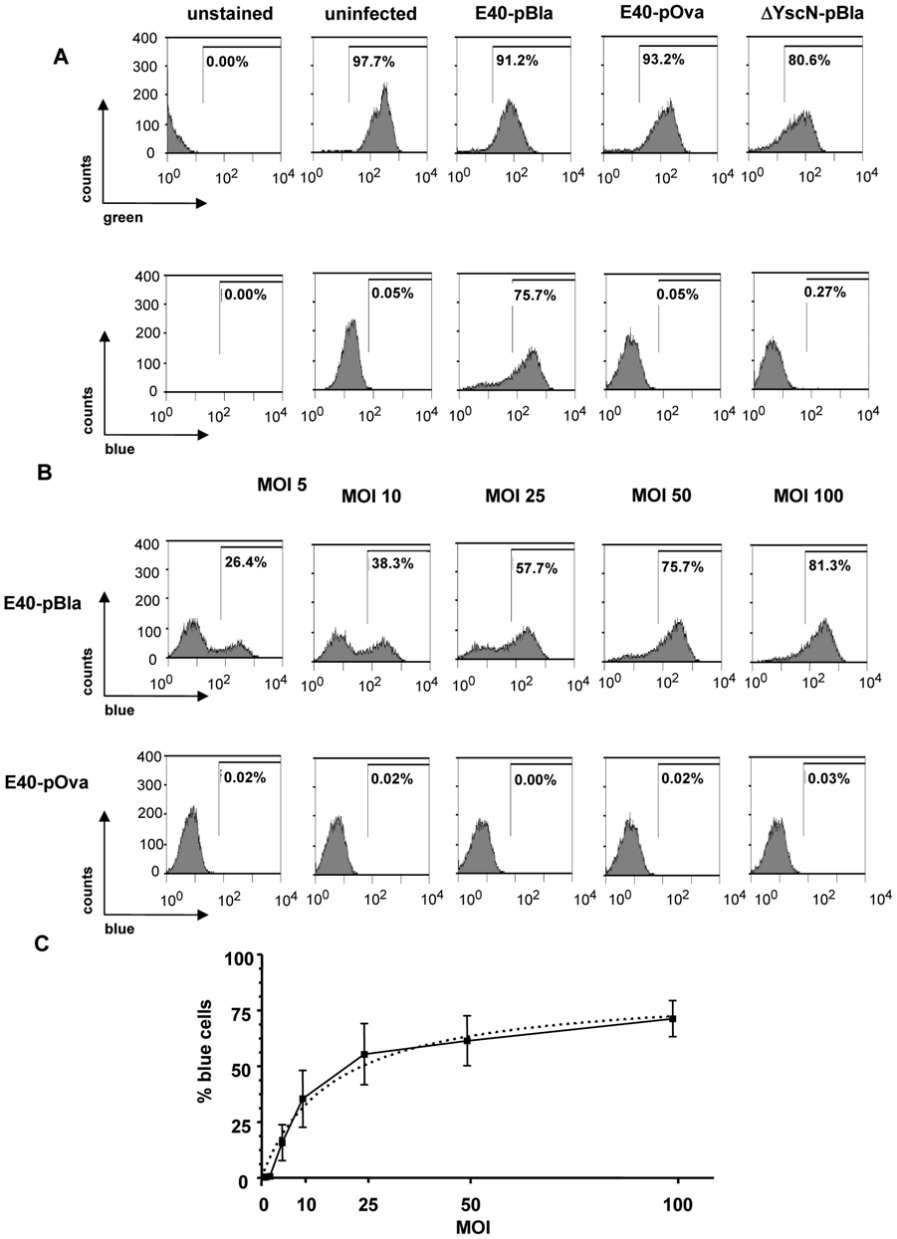
Detection of Yop injection in cultured splenocytes.

To investigate whether Yops are also injected into primary cells, single cell suspensions were prepared from the spleen of C57BL/6 mice and exposed to E40-pBla, E40-Ova or ΔYscN-pBla mutant strains at a MOI of 50 for one hour in vitro. Similar to infection of HeLa cells, flow cytometry analysis showed that CCF4 dye retention (green fluorescence) after infection of splenocytes was not significantly affected by infection with the strains used (data not shown). After infection of spleen cells for one hour with E40-pBla (MOI 50) in 61.4±11.4% of cells a blue fluorescence signal was detectable indicating that 61% of splenocytes had been injected with Yops (Figure 3A). In contrast, blue fluorescence was observed in less than 0.3% of splenocytes after infection with E40-pOva or ΔYscN-pBla. The low background of blue cells after infection with E40-pOva indicates that spontaneous cleavage of CCF4 detectable after infection with yersiniae is negligible. The low background staining after infection with ΔYscN-pBla indicates that secretion of YopE-Bla is a prerequisite for the detection of blue cells (Figure 3A) and that expression of YopE-Bla inside the bacteria and potential internalization of bacteria do not lead to significant blue fluorescence.

To determine the relationship between the number of bacteria per targeted host cell and the occurrence of blue cells (FRET disruption), spleen cell suspensions were infected with E40-pBla (and E40-pOva as a control) at various MOI for one hour and then subjected to flow cytometry analysis (Figure 3B and C). The data show that the percentage of blue cells correlates with the infection dose (MOI) in a fashion that a strong increase of the number of blue cells is followed by a saturation state. A hyperbolic regression curve can be calculated (goodness of fit of r^2^ = 0.0977) with the formula: % blue cells  =  84×MOI/(16.9 + MOI). Using this regression curve it can be calculated that an MOI of 16.9±1.9 would result in 50% blue cells. This regression curve also predicts that in cell culture even at high MOI Yop injection appears to be limited for yet unknown reasons.

**Figure 4:**
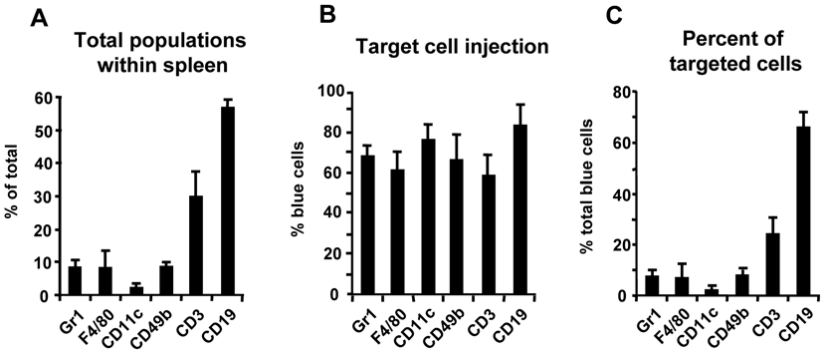
Distribution of Yop injection in cultured splenocytes. Splenocytes were infected with E40-pBla (MOI 50) for 1 h. Subsequently cells were stained with CCF4-AM as well as antibodies against indicated surface markers. (A) Percentage of splenocytes expressing the indicated surface markers. (B) Percentage of blue cells of the indicated subpopulations (C) Percentage of blue cells expressing one of the indicated surface markers. Data are summarized as the mean±SD of three independent experiments.

We also investigated whether there might be preferential Yop injection into certain cell types. To define the populations of the spleen injected with YopE53-Bla, splenocytes were infected with E40-pBla and then flow cytometry analysis was used to detect distinct cell surface markers on and β-lactamase activity in the cells. The data depicted in Figure 4A shows the composition of spleen cells and confirmed that B cells (57±2% CD19^+^) and T cells (30±7% CD3^+^) are the most abundant spleen cell subpopulations. As shown in Figure 4B, the percentage of blue cells in each analyzed spleen cell population ranged between 58±6% to 82±10% but revealed no significant differences (p>0.05) indicating that Yop injection occurs into all cells types to a similar degree. Consistently, the composition of blue spleen cells (Figure 4C) is closely related to the frequency of the subpopulations.

### Detection of Yop injection into spleen cells of Ye infected mice in vivo

**Figure 5:**
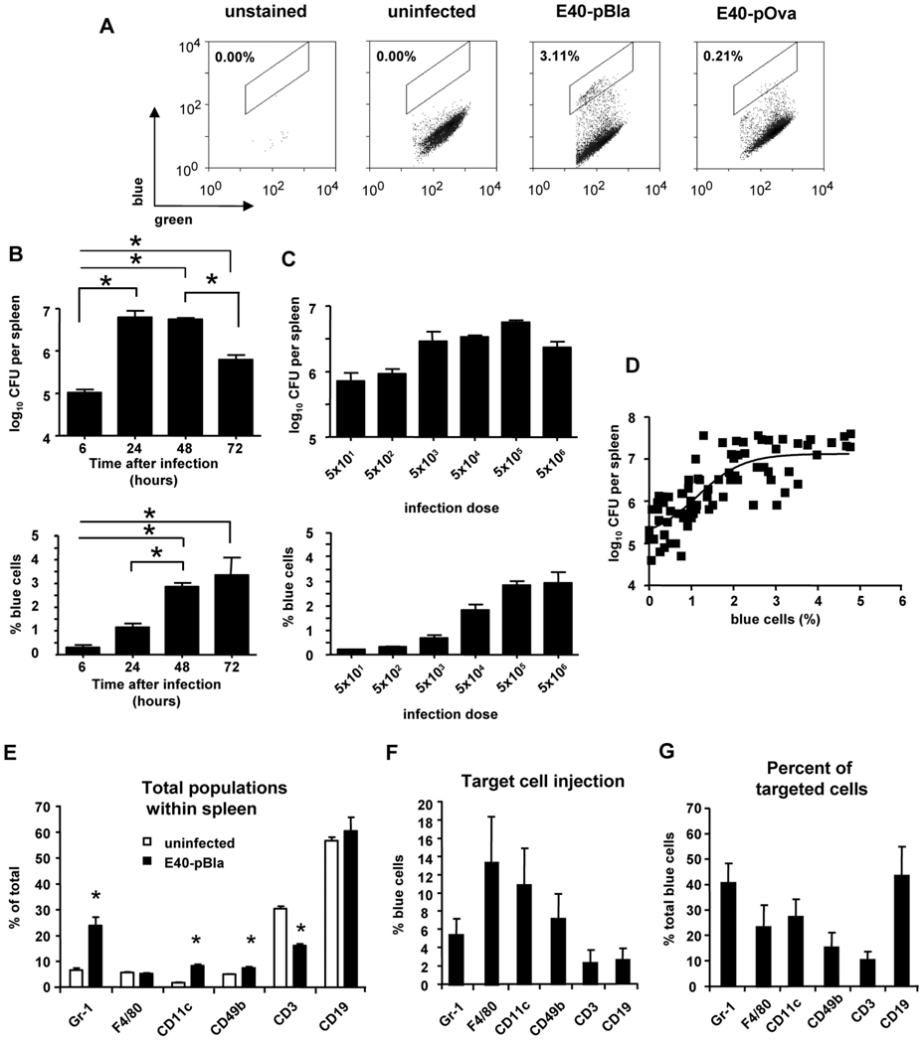
Detection of Yop injection in splenocytes derived from infected mice. Mice were infected with indicated *Yersinia* strains and isolated splenocytes were stained with CCF4-AM. In some experiments, cells were additionally stained with antibodies specific for cell surface markers (B–D). Subsequently cells were analyzed by flow cytometry. (A) Dot plots show cells gated on high levels of green fluorescence of the substrate CCF4 (viable cells) plotted versus the blue fluorescence revealing Yop-injection (infection dose 5×10^5^ for 2 days). (B) Four mice (two independent experiments with each two animals) per group were infected with 5×10^5^ E40-pBla for indicated time periods or with (C) indicated numbers of bacteria for two days and the log_10_ CFU in the spleen (upper panel) and the percentage of blue cells (lower panel) was determined. Data represent the mean±SD. (D) summarizes the correlation between the log_10_ CFU and the percentage of blue cells in the spleen of E40-pBla infected C57BL/6 mice by using data of experiments with different infection dose as well as different time ranges of infection (n  =  84). A sigmoidal regression curve was calculated (goodness of fit r^2^ = 0.64, sy.x  =  0.5). (E) summarizes the percentage of splenocytes expressing the indicated surface markers prior to and after infection with indicated *Yersinia* strains (infection dose 5×10^5^ for 2 days). Data represent the mean±SD of 5 uninfected, 5 E40-pOva or 22 E40-pBla infected mice of more than five independent experiments. (F) depicts the percentage of blue cells after infection with E40-pBla for each indicated subpopulation and (G) shows the mean±SD of the percentage of blue cells expressing one of the indicated surface markers summarizing 11 independent experiments with two mice (lowest panel), n  =  22. Comparison of all groups with each other by one-way ANOVA analysis and Bonferroni correction revealed significant differences p<0.05 in: (B) for CFU and for % blue cells: as indicated by asterisks, in (C) for CFU: 5×10^1^ *versus* 5×10^3^ 5×10^4^, 5×10^5^ or 5×10^6^ and 5×10^2^ *versus* 5×10^3^ , 5×10^4^ and 5×10^5^, for % blue cells: 5×10^1^, 5×10^2^ or 5×10^3^ *versus* 5×10^4^ , 5×10^5^, or 5×10^6^, 5×10^4^ *versus* 5×10^5^. Asterisks indicate differences in (E) if uninfected *versus* infected is compared (One-Way ANOVA with Dunnett corrections, p<0.05).

To investigate whether Yop injection can be detected in vivo, desferrioxamine-conditioned C57BL/6 mice were intravenously infected with E40-pBla or E40-pOva; one to three days later splenocyte suspensions were prepared, stained with CCF4-AM and subjected to flow cytometry analysis (Figure 5A and Figure S1). As indicated in Figure 5A and Figure S1, after infection with E40-pBla a distinct population of blue cells (3.1±1.1% of total spleen cells) could be identified which was not present in the spleen of uninfected or E40-pOva (0.2±0.2%) infected mice. To confirm data obtained by flow cytometry, cell suspensions were subjected to flow cytometry cell sorting and subsequently analyzed by fluorescence microscopy (data not shown).

Analysis of β-lactamase activity after infection for different time periods revealed a time-dependent increase of the percentage of blue cells with a maximum between two and three days after infection. The highest bacterial burden in the spleen was found between 24 and 48 hours after infection (Figure 5B). Increased infection dose led to a modest increase of the bacterial load in the spleen two days after infection (Figure 5C). However, an infection with more than 5×10^3^ E40-pBla did not further increase the bacterial burden in the spleen. The number of blue cells increased with the infection dose. In Figure 5D the bacterial load in the spleen and the percentage of blue cells from various experiments is given. The best fit to approximate the correlation of bacterial load and percentage of blue cells was obtained using a sigmoid regression curve (Figure 5D) (goodness of fit: r^2^ = 0.64, degrees of freedom  =  79, and Sy.x  =  0.50). Plotting the percentage of blue cells versus the bacterial load in the spleen also revealed that for the detection of blue cells over background (defined as % blue cells±2-fold standard deviation  =  0.6% blue cells) the bacterial load has to be more than log_10_ CFU 5.6 per spleen.

To investigate in which cells Yops were injected, C57BL/6 mice were infected with 5×10^5^ E40-pBla or E40-pOva for two days and subsequently cells were stained with CCF4-AM and fluorescence labeled antibodies binding distinct surface markers. Infection of mice with E40-pOva or E40-pBla for two days resulted in a bacterial load of log_10_ CFU 7.4±0.2 or log_10_ CFU 7.3±0.2 per spleen. Immunostaining and flow cytometry analysis of spleen cells revealed that infection with E40-pBla led to a significant change in the composition of cell populations compared to uninfected (Figure 5E). Thus, the number of Gr-1^+^ (granulocytes), CD11c^+^ (dendritic cells) and CD49^+^ (NK) cells increased significantly, while the number of CD3^+^ T cells decreased significantly. Moreover, we found that 13.4±5.1% of all F4/80^+^ (macrophages), 7.2±2.7% of all CD49^+^ (NK cells), 10.9±3.8% of all CD11c^+^ (dendritic cells) and 5.5±1.8% of all Gr-1^+^ (granulocytes) cells displayed blue fluorescence indicating that they had been injected with YopE53-Bla (Figure 5F). In contrast, only 2.3±1.3% of all CD3^+^ (T cells) and 2.6±1.1% of all CD19^+^ (B cells) cells displayed blue fluorescence. These data suggest that Yop injection upon Ye infection in this mouse infection model occurred predominantly into cells of the myeloid lineage representing the innate immune system. Analysis of the composition of total spleen cells with blue fluorescence regarding the percentage of each subpopulation of cells revealed that related to the total number of blue cells 43.8±11.3% were CD19^+^, 40.8±7.6% were Gr-1^+^, 26.9±3.7% were CD11c^+^, 23.6±8% were F4/80^+^, 15.4±5.3% were CD49b^+^, and 10.4±3 were CD3^+^. These data indicate that injection of Yops occurred into both myeloid and lymphoid cells, most frequently into B cells and granulocytes (Figure 5G).

### Increased activation of Yop-injected B cells

Splenic B cell subpopulations comprise CD19^+^CD21^hi^CD23^−^ (marginal zone B cells, MZ), CD19^+^CD21^+^CD23^+^ (follicular B cells, FO), and CD19^+^CD21^lo^ (newly formed B cells, NFB) B cells. To address whether Yop injection occurs in all of these B cell subpopulations, cultured splenocytes were infected in vitro with E40-pBla (MOI 50) for 1 hour and Yop injection was determined after staining using antibodies for B cell markers and CCF4 (Figure S2). The data show ∼84% of B cells were blue indicating injection of Yops; moreover, the different B cell subpopulations contributed to the total number of CD19^+^ blue cells according to their different frequencies of MZ (∼10%), FO (∼70%) and NFB (∼20%), as injection of Yops occurred with similar efficacy in all B cell subtypes in vitro.

**Figure 6:**
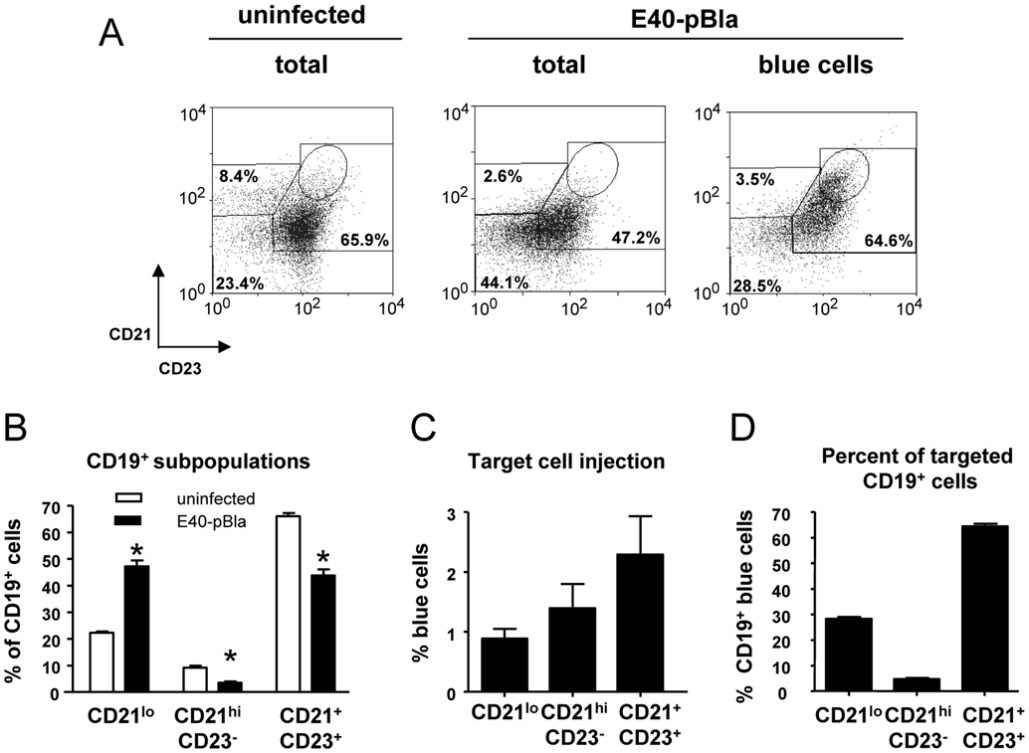
Determination of Yop injection in B cell populations. (A) Desferrioxamine conditioned mice were mock-infected or were infected with 5×10^5^ E40-pBla for two days. Splenocytes were then stained with CD19-APC, CD21-PE-Cy7 and CD23-APC-Cy7 and with CCF-AM and then analyzed by flow cytometry. Cells were gated for viable CD19^+^ cells and then analyzed for expression of blue and green fluorescence as described in Figure S2. All viable CD19^+^ cells (total) or blue fluorescent CD19^+^ cells were then analyzed for CD21 and CD23 expression. CD21^hi^CD23^−^ cells were defined as MZ (marginal zone B cells), CD21^+^CD23^+^ as FO (follicular B cells) and CD21^−^ as NFB (newly formed B cells). In each diagram 10000 events are depicted. The percentage of each of these populations is indicated showing a representative experiment and percentages of the subpopulation are indicated for this experiment. (B) depicts the distribution of MZ, FO and NFB of CD19^+^ cells prior to and after infection of mice with E40-pBla. Asterisks indicate significant differences compared to uninfected (p<0.05) (C) Mean and SEM of the percentage of blue cells for each CD19^+^ subpopulation. (D) shows the mean and SEM of the distribution of each subpopulation of all blue fluorescent CD19^+^ cells. In total four mice per group in two independent experiments were analyzed.

To analyze Yop injection into B cells in vivo, desferrioxamine-conditioned C57BL/6 mice were infected with 5×10^5^ E40-pBla and two days later B cells were analyzed (Figure 6A). After infection, the composition of splenic B cell subpopulations changed. Thus, the percentage of CD21^+^CD23^+^ (FO) (43.8±4 in infected versus 66%±2 in uninfected mice) and the percentage of CD21^hi^CD23^−^ (MZ) (3.6±0.9 in infected versus 9.2±1.4 in uninfected mice) significantly decreased whereas the percentage of CD21^lo^ (NFB) (47.2±3.8 in infected versus 22.2±0.9 in uninfected mice) significantly increased. Taking the changes in total cell numbers in the spleen into account, the total number of NFB significantly increased while the total number of FO and MZ remained largely unchanged (data not shown). Analysis of blue (Yop-injected) B cells (∼2% of total B cells) revealed that 64.4%±1.7 of CD19^+^ blue B cells were CD21^+^CD23^+^ (FO) B cells indicating that *Yersinia* targets predominantly follicular B cells (Figure 6C). In blue FO B cells, a distinct CD21^hi^CD23^hi^ B cell subpopulation (Figure 6D) was detected suggesting that injection of Yops into FO is associated with an increased expression of CD21 and CD23.

**Figure 7:**
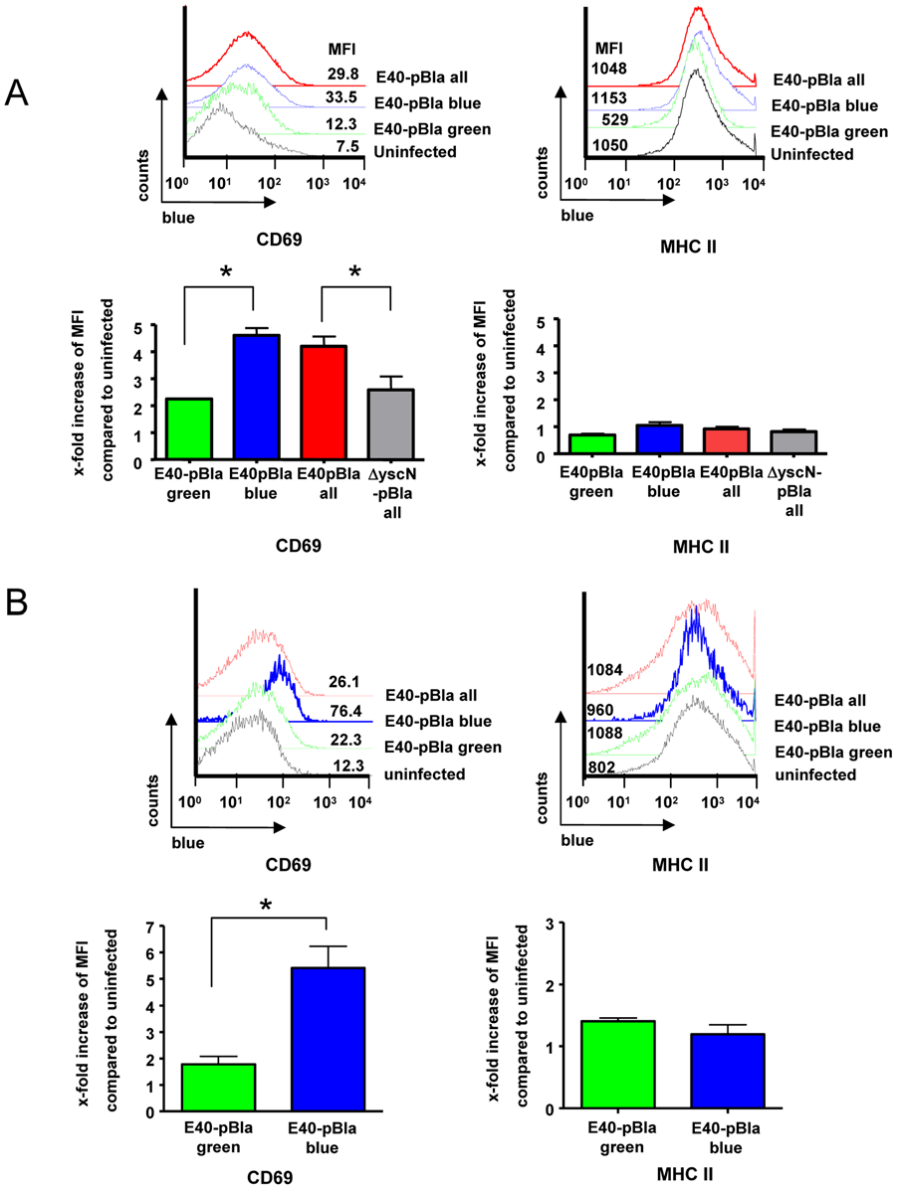
Changes of CD69 expression in splenocyte populations after *Yersinia* infection. (A) Cultured splenocytes were infected with E40-pBla (MOI 50) or mock-infected for 1 hour or (B) desferrioxamine conditioned C57BL/6 mice were left untreated or infected with 5×10^5^ E40-pBla for two days and the splenocytes were then isolated. Splenocytes were stained with anti-CD19-APC, anti-CD69-PE-Cy7 and subsequently with CCF4 and analyzed by flow cytometry. Cells were gated (as shown in Figure S3) for high green fluorescence (viable cells) and B cells (CD19^+^). Left panels show histograms for CD69 expression of non-infected cells gated for viable CD19^+^ cells (black line) or of E40-pBla infected cells gated for all viable CD19^+^ cells (total, red line), gated for green^+^ blue^+^ CD19^+^ cells (blue line) or green^+^ blue^−^ CD19^+^ cells (green line). Mean fluorescence intensities (MFI) of CD69 expression are depicted for this representative experiment. Right panels show the means and SEM of the x-fold increase of MFI of the indicated cells compared to uninfected of (A) three independent experiments or (B) two independent experiments with a total of five mice per group. Asterisks indicate significant differences, p<0.01.

To address whether injection of Yops may affect activation of B cells, expression of CD69, an early activation marker of B cells, was determined. In splenocytes infected in vitro with E40-pBla, increased CD69 expression was found in B cells (mean fluorescence intensity, MFI; 7.5 in uninfected versus 29.8 in infected cultures). Moreover, in Yop-injected B cells (green^+^ blue^+^), expression of CD69 was higher compared with B cells not injected with Yops (green^+^ blue^−^) (Figure 7A) suggesting that in vitro, injection of Yops into B cells is associated with rapid activation of these cells.

Upon infection of mice with E40-pBla, ∼2% of B cells were injected with Yops (blue staining) (Figure 7B). Expression of CD69 was increased in splenic B cells compared with uninfected mice (MFI of 26.1 in infected versus 12.3 in uninfected mice). Moreover, in Yop-injected B cells (green^+^ blue^+^), expression of CD69 was dramatically higher (MFI 76.4) compared with B cells not injected with Yops (green^+^ blue^−^) (MFI 22.3). In conclusion, these data suggest that direct interaction of Ye with B cells including injection of Yops leads to activation of B cells as indicated by increased CD69 expression.

### Investigation of Yop injection after infection of TNF-Rp55 and IFN-γR deficient mice

**Figure 8:**
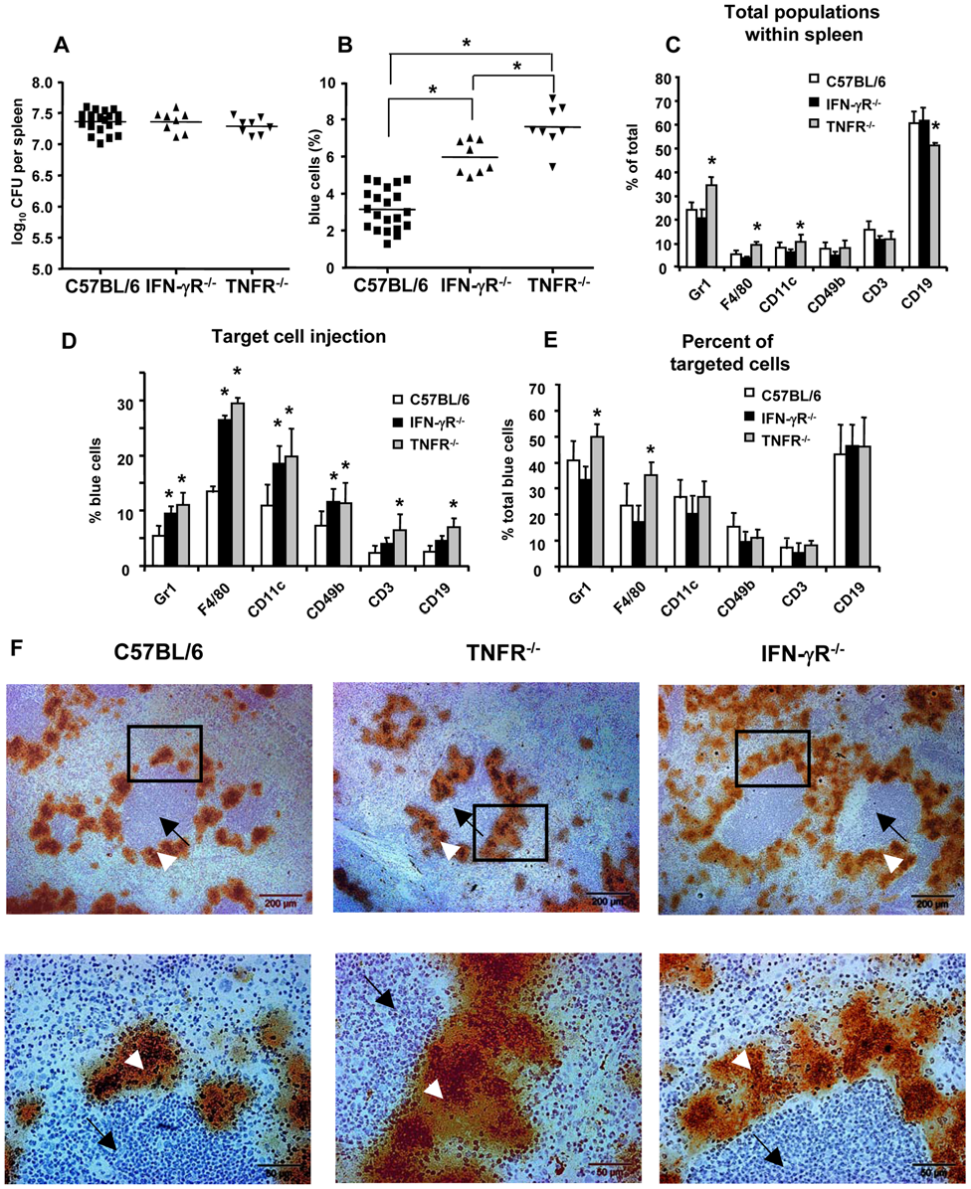
Impact of IFN-γR and TNF-R deficiency on Yop injection. IFN-γR^−/−^, TNFRp55^−/−^ and control C57BL/6 mice were infected with E40-pBla and two days later (A) bacterial load in the spleen and (B) percentage of blue cells was determined. Each dot depicts the results for one single mouse (in total 8–22 mice were investigated). Data in (C) show the mean and SD of the distribution of cell subpopulations expressing indicated markers, in (D) the mean and SD of percentage of blue cells of each indicated subpopulation and in (E) the mean and SD of the percentage of the indicated subpopulations of blue cells. Asterisks show significant differences between all groups compared (A, B, one-way ANOVA with Bonferroni corrections, p<0.05) and differences between control mice and knockout mice (C, D, E; one-way ANOVA, with Dunnett corrections, p<0.05). F. Immunohistology of infected spleen of indicated mice. Splenic sections were stained with anti-hsp60 and counterstained with Mayer's hemalaun. White arrowheads mark lymph follicle and black arrows colonies of yersiniae. All the experiments were repeated more than four times using in total 8–22 mice. Marked regions are shown at higher magnification in lower panels.

Previous studies showed that TNFRp55^−/−^ and IFN-γR^−/−^ mice are highly susceptible to infection with Ye [36]–[40] indicating that the pleiotropic effects of IFN-γ and TNF-α are crucial for the defense against yersiniae. To investigate whether such gene defects may modulate Yop injection, desferrioxamine conditioned TNFRp55^−/−^, IFN-γR^−/−^, and wild type mice were infected with Ye and two days later, splenocytes suspensions were prepared, stained with antibodies and CCF4-AM, and subjected to flow cytometry analysis. Infection of TNFRp55^−/−^ and IFN-γR^−/−^ mice with E40-pOva as well as E40-pBla resulted in a bacterial load of log_10_CFU 7.4±0.1 and 7.3±0.1, respectively and was comparable to the bacterial load of infected WT mice (log_10_CFU 7.4±0.1) (Figure 8A).

After infection of IFN-γR*^−/−^* mice no significant changes in the splenic cell subpopulations were observed (Figure 8C) compared to infection of WT mice. However, the percentage of all blue cells increased significantly (IFN-γR^−/−^ 5.9±0.9% versus WT 3.1±1.1%) (Figure 8B). Likewise, the percentage of blue Gr-1^+^, F4/80^+^, CD11c^+^ and CD49b^+^, but not CD3^+^ and CD19^+^, cells increased significantly (Figure 8D). Taking both, the total number of blue cells as well as changes in the cell composition into account, no significant alterations in the composition of blue cells was found (Figure 8E). These data indicate that IFN-γR deficiency leads to an increased number of cells, in which Yops are injected, but the composition of blue cells is comparable with that of infected C57BL/6 mice.

Infection of TNF-Rp55^−/−^ mice led to a significant increase in the percentage of Gr-1^+^, F4/80^+^ and CD11c^+^ cells, and a significant decrease of C19^+^ cells compared with WT mice (Figure 8C). The percentage of total blue cells was significantly higher after infection of TNF-Rp55^−/−^ (7.6±1.1%, p<0.001) compared with infection of WT mice (3.1±1.1%) (Figure 8B). In line with these findings, all subpopulations (F4/80, Gr-1, CD11c, CD49b, CD19 and CD3) showed a significantly increased percentage of blue cells in infected TNFRp55^−/−^ mice (Figure 8D). The composition of blue cells changed leading to a significantly increased number of blue Gr-1^+^ and F4/80^+^ cells compared to WT mice (Figure 8E). These data suggest that TNFRp55 deficiency leads to a more frequent injection of Yops into F4/80^+^ and Gr-1^+^ cells.

To investigate the localization of yersiniae in the spleen after infection of mice, splenic sections were prepared and immunochemistry using anti-*Yersinia* Hsp60 antibodies was performed (Figure 8F). Histological and immunohistological studies revealed that Ye formed many microcolonies surrounding the lymphoid follicles. Gross differences in the splenic architecture or localization of yersiniae in the spleen of WT or knockout mice were not observed.

To further address why in *TNFRp55* ^−/−^ and *IFN-γR* ^−/−^ mice increased numbers of Yop injected spleen cells were found, splenocytes of C57BL/6, TNFRp55^−/−^ and IFN-γR^−/−^ mice were infected in vitro for one hour with different MOI of E40-pBla and the percentage of blue cells was determined. The frequency of blue cells for the different MOIs was not significantly different between C57BL/6 and TNFRp55^−/−^ or IFN-γR^−/−^ splenocytes (Figure S4) suggesting that splenocytes of wildtype, TNFRp55^−/−^ and IFN-γR^−/−^ mice upon infection with Ye display a comparable frequency of Yop injection.

## Discussion

Injection of Yop effector molecules into host cells by the TTSS is an important strategy of Ye to suppress the host immune response. However, detection of Yop injection in animal infection models has been difficult so far. Several tools have been employed to detect injection of bacterial proteins into host cells, but their usefulness has been limited to certain in vitro models [8], [25]–[28]. In similar, results published by Briones et al. and own attempts to create a Cre-recombinase based reporter system to monitor Yop injection into host cells produced promising results in cell culture experiments but failed to deliver a valuable tool in mouse infection models [29]. Recently however, Marketon *et al.* demonstrated that Yop-β-lactamase fusion proteins can be utilized to monitor Yop injection in vivo [30]. In this study we applied this tool to Ye infection and tested whether it might be useful to detect Yop injection in vitro and in vivo.

In line with the report by Marketon *et al.* [30], the use of control strains such as ΔYscN-pBla and E40-pOva revealed that detection of β-lactamase reporter activity depends on injection of the YopE53-Bla fusion protein. Therefore, internalization of bacteria does not account for reporter activity. Yop injection into host cells was detectable after infection of HeLa epithelial cells and primary C57BL/6 splenocytes in cell culture experiments. In vitro experiments with splenic subpopulations indicated that Yop injection occurs with similar efficacy into macrophages, dendritic cells, granulocytes, NK cells, T cells or B cells in cell culture. We conclude that the different immune cell subpopulations display the same prerequisites for Yop injection. From these data it is conceivable that Yop injection in vivo actually reflects the interaction frequency of bacteria with distinct splenic subpopulations.

The usefulness of this reporter system in cell culture experiments could be demonstrated by investigating the importance of β1-integrins for Yop injection. It is widely accepted that β1-integrins play an important role for adhesion to host cells and eventually for internalization of yersiniae via the direct or indirect interaction with invasin and YadA, respectively [34], [41]–[43]. In line with this, the number of adhering Ye to fibroblasts which lack β1-integrins was reduced by 40% compared to cells expressing β1-integrins. In contrast, Yop injection (measured as percentage of blue fluorescent cells) into fibroblasts which lack β1-integrins was dramatically reduced by 95% compared to fibroblasts expressing β1-integrins. These data are in line with a recent report [35] which suggested a central role for β-integrins for Yop injection. Nevertheless, detection of YopE translocation by immunoblots was still detectable in GD25 cells which lack β1-integrins, indicating that other surface molecules beside β1-integrins may also play a role to modulate Yop injection. At this point it is not clear which other surface molecules beside β1-integrins affect Yop injection and whether depending on the cell types in which Yops are injected different surface molecules modulate Yop injection in vitro and in vivo.

After *Y. pseudotuberculosis* infection β1-integrin mediated signal transduction leading to RhoGTPase activation seems to be the prerequisite for initiation of Yop effector injection [35]. By using the Rac1 inhibitor NSC23766 it was excluded that Rac1 plays a role for Yop injection and because both TcdB (inhibitor of RhoA, Rac1, Cdc42, RhoG, Tc10) and C3 exotoxin (RhoA, RhoB, RhoC inhibitor) inhibited Yop injection, RhoA, RhoB and RhoC were identified as putative candidates for this effect [35]. In contrast, the siRNA experiments presented herein suggest that both Rac1 and RhoA play a role for Yop injection after Ye infection of HeLa cells. Interestingly, in the present study the Rac1 inhibitor NSC23766 also did not inhibit Yop injection. NSC23766 was demonstrated to bind to Rac1 and to prevent binding to and activation of Rac1 by the Rac1-specific guanine nucleotide exchange factors (GEF) Tiam1 and Trio. However, it was described that NSC23766 seems not to prevent binding to and activation of Rac1 by the more promiscuous GEF Vav [44],[45]. As Vav is able to activate both RhoA and Rac1, but not Cdc42 [44],[45], one may speculate that Vav or other GEFs, rather than Tiam1 and Trio, might be involved in Rac1 mediated Yop injection. One has to anticipate that NSC23766 inhibits *Yersinia* triggered Rac1 activation only partially and in a GEF specific manner which has to be proven in future studies. In addition it will be interesting to find out which specific GEFs are involved in facilitating Yop injection. Taken together this study clearly pinpoints that β1-integrins play a role in facilitating Yop injection.

The YopE-Bla reporter system turned out to work highly efficiently when expressed in E40 (O9) strain, but not in WA-314 (O8) strain; thus, 80% of in vitro infected splenocytes displayed blue fluorescence upon infection with E40-pBla strain, but only 26% upon infection with WA-pBla strain (data not shown). Moreover, in C57BL/6 mice infected with E40-pBla 3% of splenocytes displayed blue fluorescence, while in WA-pBla infected mice we could hardly detect any blue cells for yet unknown reasons. We therefore used the E40 strain for all experiments included in this study. The low virulence of serotype O9 strains such as E40 strain or O3 strains in mice is at least partially caused by their disability to synthesize the iron chelating siderophore yersiniabactin due to their lack of the HPI island [46],[47]. This could be “complemented” by treatment of mice with desferrioxamine which allows to study Ye O9 and Ye O3 infection in mice [48]–[50]. The disadvantage of desferrioxamine, however, is that it might have immunosuppressive effects on phagocytes [51],[52].

Infection of desferrioxamine-conditioned C57BL/6 mice revealed a correlation between the percentage of blue cells and the bacterial burden in the spleen which can be best described by a sigmoidal dose response curve. Taking the background staining of 0.2±0.2% into account, a detection limit of 0.6% blue cells was estimated using the sigmoidal regression curve. To reach 0.6% blue cells in the spleen the bacterial burden would have to be at least 4×10^5^ bacteria representing a MOI of 0.01. The cell culture experiments, however, showed that much higher MOI are required to achieve half-maximal numbers of blue spleen cells. Therefore, higher efficacy of YopE-bla detection might be due to abscess formation with Ye microcolonies which may allow more Ye bacteria to interact with single host cells at abscesses. Alternatively the TTSS machinery might be upregulated during infection in spleen compared to cell culture. Most experiments were carried out at conditions with a bacterial burden of 5×10^7^ bacteria in the spleen resulting in 3.1±1.1% of blue, Yop-injected cells.

Determination of the percentage of blue cells in each subpopulation revealed that Yop injection occurred into Gr1^+^ (predominantly granulocytes), CD11c^+^ (predominantly dendritic cells), F4/80^+^ (predominantly macrophages) and CD49b^+^ cells (predominantly NK cells). In contrast, only in a small percentage of the CD3^+^ (T cells) and CD19^+^ (B cells) cells, Yop injection was detectable. However, due to the high total number of B cells (>50% of spleen cells) their contribution to the total number of blue cells is about 40%. Further characterization of B cells revealed that the composition of B cell subpopulations changed after infection leading to an increased number of CD19^+^CD21^lo^ (NFB) while the number of CD19^+^CD23^+^CD21^+^ cells (FO) did not change and the number of CD19^+^CD21^hi^ CD23^−^ (MZ) slightly decreased. Thus, after infection NFB appear to either proliferate in or immigrate into the spleen.

Yop injection was predominantly detectable in follicular B cells and to a lesser extent in NFB.

Naïve follicular B cells reside in the “follicular niche” and may present T-dependent antigens to activated T cells. The follicular niche therefore represents the major site at which recirculating B cells mediate T-dependent immune responses to protein antigens [53]. Interestingly, in contrast to the Ye infection model presented here, injection of the Bla-reporter into B cells was not described in *Y. pestis* infection [30]. It remains unclear whether these discrepancies are due to different *Yersinia* species, different mouse strains or differences in the reporter systems. Nevertheless, it is striking that after *Y. pestis* infection the analysis gate which was used to define the viable cells was rather small indicating that most spleen cells were necrotic or apoptotic under the chosen experimental conditions. It might well be that Yop-injected B cells might have been missed by the analysis due to high cell death rates in *Y. pestis* infection.

In Ye infection, however, interaction of B cells with Ye is prominent. This finding is in line with the analysis of histological sections of the spleen. Thus, E40-pBla microcolonies were observed adjacent to lymphoid follicles (B cell zones). Balada-Llasat and Mecsas [54] reported that *Y. pseudotuberculosis* may show a tropism to B and T cell zones in lymph nodes. In our studies we found yersiniae always adjacent to lymph follicles in the spleen and the most frequent interaction of yersiniae with host cells was that with B cells, specifically with CD19^+^CD21^+^CD23^+^ B cells which are defined as follicular B cells (FO). FO B cells organize into the primary follicles of B cell zones focused around follicular dendritic cells in the white pulp of the spleen. Thus in our system Ye appear to preferentially colonize in the follicular niche and interact frequently with follicular B cells. Whether Ye are actively migrating to this region or may be trapped by e.g. dendritic cells needs to be further investigated. Infection with Ye was associated with increased CD21 and CD23 expression levels of FO B cells which suggests that B cells may be activated after interaction with yersiniae. Specific activation of Yop injected B cells was also supported by the observation that the early B cell activation marker CD69 was upregulated in blue B cells. From this data we can conclude that Yop-injected B cells were specifically activated by Ye despite injection of Yops. Whether Yop-injected B cells may undergo subsequent cell death or are affected in their ability to produce IgM or to undergo differentiation needs to be investigated in future study.

Previous studies revealed that TNF-α and IFN-γ are important for defense against Ye infection [36]–[38],[40]. Therefore, we were interested whether these cytokines may affect Yop injection. The experimental conditions were chosen in a way that the bacterial burden in the spleen of the various mouse strains was comparable. Infection of IFN-γR- and TNFRp55-deficient mice revealed a higher number of blue cells in the spleen compared to infected WT mice indicating that either more host cells interacted with yersiniae or the threshold which allows detection of Yop injection was lower in knock-out mice. Histological analysis of the Ye microcolonies in WT and knockout mice revealed comparable tissue distribution of Ye and comparable tissue alterations by the infection. In vitro infection of splenocytes from IFN-γR- and TNFRp55 deficient and wildtype mice did not reveal significant differences in terms of Yop injection. Therefore it remains widely unclear why more blue cells are found in IFN-γR- or TNFRp55^−/−^ mice compared to wild type mice. However, we cannot exclude that minor changes in spleen cell subpopulations may partially cause the different numbers in knock-out and wild type mice.

In further experiments we tried to detect Yop injection into cells of the PP after orogastric infection. However, we were not able to detect blue PP cells under these conditions (data not shown). One explanation might be that the number of bacteria that establish infection in the PPs is lower than that in spleen [55]. Thus, one may speculate that the number of bacteria attached to a single host cell in PP might be lower than that in spleen which might result in Yop injection below the detection limit of this reporter system. Alternatively or in addition, the number of cells in which Yops were translocated might be too low.

The Bla reporter system described herein is so far the only reporter system that allows tracking of Yop injection in Ye infection in vivo on a single cell level in a mouse infection model. Nevertheless, the system needs to be improved in order to accomplish a more sensitive reporter system. The disadvantages of CCF-4 as a substrate for β-lactamase is that the amount of Yops translocated cannot be quantified and that CCF-4 displays limited sensitivity. Moreover, CCF-4 does not allow studying Yop injection in histological sections in situ. Current experiments in our laboratory aim at overcoming these limitations with new compounds.

Taken together, the Ye Bla reporter system can be successfully used in cell culture and in mouse infection models for detection of Yop injection on a single cell level, and thus could be a valuable tool for quantitatively screening for genetic factors involved in Yop secretion and injection at the bacterial as well as at the host cell side, or to identify drugs which target Yop secretion or injection, respectively.

## Materials and Methods

### Bacterial strains and growth conditions

*Y. enterocolitica* E40 strains used in this study are listed in table 1. All bacterial strains were grown overnight in Luria-Bertani (LB) broth at 27°C supplemented with 50 µg/ml meso-diaminopimelic acid, 50 µg/ml nalidixic acid, 50 µg/ml kanamycin, and 400 µM Na-arsenite (all from Sigma Chemical, St. Louis, MO) in combinations according to the indicated resistances and supplementation needs (table 1). A 1∶25 dilution of the bacterial culture was incubated for additional 3 h at 37°C. The bacteria were washed once with phosphate-buffered saline (PBS; Invitrogen, Karlsruhe, Germany) and the optical density at 600 nm was determined.

### Plasmid constructions

All plasmid constructs and bacteria used are listed in table 1. To generate pMK-Bla, as a starting vector pBME53-yopT [56], a pACYC184 derivate containing a *Hind*III-*sycE*-*yopE*/*sycE* promoter-*yopE* gene fragment encoding the first 53 N-terminal amino acids (aa) -*Bam*HI-YopT -*Sal*I construct was used. YopT was replaced by a translational fusion of the coding sequences (CDS) of the SV40 nuclear localization signal (nls) and the CDS of Cre recombinase resulting in pBME53-Cre. This vector was digested with *Hind*III and *Sal*I and the resulting *sycE*-*yopE53*-*cre* fragment was inserted into pIV2 [57]. pIV2 was derived from a small cryptic plasmid of an apathogenic *Y. enterocolitica* strain (kindly provided by Eckhard Strauch, Federal Institute for Risk Assessment, Berlin) resulting in pIV2-SycE-YopE53-Cre. To create a balanced system that compels stable retention of the reporter vector in *Y. enterocolitica* during in vivo infections, the *Y. enterocolitica asd* gene coding for the L-aspartate-dehydrogenase, an enzyme that is required for the synthesis of the cell wall component L-lysine via the *meso*-2,6-diaminopimelic acid (DAP) pathway and therefore essential for bacterial growth and replication was inserted into the pIV2-SycE-YopE53-Cre plasmid as follows: The *asd* gene and its 5′region were amplified from *Y. enterocolitica* E40 chromosome by using the oligonucleotides 5′- AGC TTT AGG GCC CAA AAA CAG CAA CAC CGT TGC C-3′ and 5′-AAC TCG AGT TAC AGA AAA TTC GCA GC-3′. The PCR product was then digested with *Apa*I and *Xho*I and inserted into those sites of pIV2-SycE-YopE53-Cre, thus yielding pMK4. Together with the deletion of the *asd* gene from its chromosomal locus (see below) in the utilized bacteria, this grants stable retention of the reporter plasmid, by complementing the otherwise lethal DAP auxotrophy of the *Y. enterocolitica* E40 *Δasd* strain.

The β-*lactamase* (*bla*) gene was amplified from pCR2.1 (Invitrogen, Karlsruhe, Germany) using the primers 5′-GGA TCC ATG AGT ATT CAA CAT TTC CG-3′ and 5′-GTC GAC AAC TTG GTC TGA CAG TTA CC-3′. The PCR product was subcloned into pCR-Blunt II and subsequently donated as a *Bam*HI / *Sal*I fragment to pBME53-YopT, thus replacing the sequence coding for YopT and fusing the translation product to the YopE53 domain, yielding the plasmid pBME53-Bla. pBME53-Bla was digested with *Hind*III-*Sal*I and the resulting *Hind*III-*sycE*-*yopE53*-*bla*-*Sal*I fragment replaced the *Hind*III-*sycE*-*yopE53*-*cre*-*Sal*I fragment of pMK4 yielding pMK-Bla.

To generate a control vector, the sequence coding for β-*lactamase* was excised by *Bam*HI / *Sal*I digestion from pBME53-bla and replaced by a *Bam*HI / *Sal*I fragment from pYopE_1–138_Ova_247–355_ [58] encoding an internal ovalbumin fragment comprising aa 247–355, yielding pBME53-ova. From this plasmid, a fragment encoding the YopE53-Ova247–355 fusion protein was yielded by *Hind*III / *Sal*I digest and used to replace the *Hind*III / *Sal*I fragment encoding YopE53-Bla in pMK-Bla yielding pMK-Ova.

*Generation of Y. enterocolitica strains* - *Y. enterocolitica* E40 is a clinical isolate that belongs to the serotype O:9 [27]. To generate *Y. enterocolitica* E40 *Δasd* the *asd* gene in this strain was disrupted by allelic exchange. The pMK3 asd mutator plasmid was constructed as follows: (i) the 5′end of the asd gene was amplified from *Y. enterocolitica* E40 by using the oligonucleotides 5′-GAT CGT CGA CAT GGT CGG CTC AGT A-3′and 5′- CAG TGA ATT CCG GCG TCC AAT CCA ATA-3′ and the 3′end by using the oligonucleotides 5′-GAT CTC TAG ATT CGC AGC ATA CGG C-3′and 5′-GAC TGA ATT CGT GAC TGC GGC CAC T-3′. The PCR product corresponding to the 5′ end of *asd* was digested with *Sal*I-*Eco*RI and that corresponding to the 3′ end with *Eco*RI-*Xba*I. The restricted PCR products were then ligated into pBluescript II SK (+) (Stratagene, Cedar Creek, USA) digested with *Sal*I-*Xba*I, which originated pMK1 that contains a deleted asd gene (Δ*asd*). The Δ*asd* allele was subcloned as a *Sal*I-*Xba*I DNA fragment into pMRS101 [59] digested with the same enzymes, yielding pMK2. The *asd* mutator plasmid pMK3 was obtained after digestion of pMK2 with *Not*I and religation of the vector.

The strains E40-pBla and E40-pOva have been generated by electroporation of pMK-Bla or pMK-Ova, respectively into *Y. enterocolitica* E40 *Δasd*. Thus, the DAP auxotrophy of these strains was supplemented, thereby creating a metabolically balanced system assuring stable plasmid retention.

To enable the generation of reporter strains with pYV40 virulence plasmids that had been subjected to mutagenesis previously, pYV40 was cured from *Y. enterocolitica* E40-pBla as described previously [60]. Screening for arsenite susceptible clones yielded *Y. enterocolitica* E40 *Δasd pYV^−^* pMK-Bla (pYV^−^ *Δasd* pBla).

*Y. enterocolitica* E40 *Δasd yscNΔ169–177* (ΔYscN-pBla) was created by electroporation of pMSL41 [27],[61] into pYV^−^ *Δasd* pBla. The plasmid pMSL41 has been yielded by deletion of *yscN*169–177 from pYV40.

### Analyses of Yop secretion

Secretion of the YopE53-β-lactamase fusion protein was examined by the preparation of released proteins as described previously [62]. Overnight cultures grown at 27°C were diluted 1∶20 into brain heart infusion broth (Difco, Heidelberg, Germany). After 2 h of incubation at 37°C, Yop secretion was induced by the addition of 5 mM EGTA for Ca^2+^ sequestration, 15 mM MgCl_2_ and 0.2% glucose. 3 h after induction secreted proteins were precipitated from the culture supernatant with trichloroacetic acid. Protein concentration was determined by Bradford assay (Bio-Rad, Hercules, USA) and 15 µg of protein was loaded on a 12% SDS-PAGE. Separated proteins were transferred electrophoretically to an Immobilon-P PVDF membrane (Millipore, Bedford, USA). For immunostaining, polyclonal rabbit anti-YopE was used. Immunoreactive bands were visualized by incubation with peroxidase-conjugated swine anti-rabbit IgG antibody (1∶1000) (DAKO, Glostrup, Denmark) using enhanced chemiluminescence reagents (ECL, Amersham Biosciences, Freiburg, Germany) and CL-XPosure Film (Thermo, Rockford, IL).

### Cell culture and in vitro infections

The fibroblast-like GD25 and GD25-ß1A cell lines were a kind gift from R. Fässler (Max-Planck-Institute for Biochemistry, Martinsried, Germany). The murine GD25 cells were derived from the embryonic stem cell clone G201 which is deficient in the ß1-integrin subunit [63]. The stably transformed cell line GD25-ß1A was obtained by electroporation of wild-type integrin ß1A cDNA into GD25 cells [64]. GD25 cells not expressing ß1-integins were cultivated in DMEM (Gibco) supplemented with 10% FCS , the GD25-ß1A line expressing ß1-integrins was maintained in DMEM supplemented with 10% FCS and 10 µg/ml puromycin.

HeLa cervical epithelial cells (ATCC CCL-2.1) were grown in RPMI 1640 (Biochrom KG, Berlin, Germany) supplemented with 10% fetal bovine serum (Sigma Chemical), 2 mM L-glutamine (Biochrom KG), penicillin (100 U/ml), and streptomycin (100 µg/ml) (Biochrom KG) in a humidified 5% CO_2_ atmosphere at 37°C. Suspensions of single spleen cells were prepared from naïve C57BL/6 mice as described below. For infection with a MOI of 100 for microscopic observation and Western Blot analysis or with a MOI of 50 or as indicated for FACS analysis bacteria were spun onto the cells (5 minutes, 400 g). After 1 h incubation at 37°C, gentamicin (100 µg/ml) was added to terminate infection. To assess the impact of inhibitors of RhoGTPases on Yop injection, cells were treated with 200 ng/ml medium *Clostridium difficile* toxin B 10463 (TcdB; kind gift I. Just, Institute for Toxicology, Hannover Medical School, Hannover, Germany) 2 h prior to infection or 100 µM Rac1 inhibitor (NSC23766; Calbiochem, San Diego, CA) 3 h prior to infection.

### Western Blot analysis of Yop injection

To analyze Yop injection by immunoblotting, cells were grown in 94 mm dishes, and then were left untreated uninfected or were infected with the E40-pBla reporter strain or the ΔYscN-pBla strain (MOI 50) as a control for 90 minutes. Cells were lysed in 200 µl PBS with 1% Triton-X (Sigma). 100 µg cell lysate protein was analyzed by SDS-PAGE and immunoblotting was performed in similar as already described for detection of Yop secretion with the exception that HRP Substrate Plus (P. J. K., Kleinbittersdorf, Germany) was used instead of ECL and in some cases film exposure was extended to 30 min for increased sensitivity.

### Adhesion to cultured cells

To assay adhesion of the E40-pBla reporter strain to HeLa, GD25 and GD25-ß1A cells, the respective cells were seeded on coverslips in a 24-well plate (Nunc Life Technologies, Wiesbaden, Germany). One hour after infection (MOI 50), cells were washed with PBS, fixed with 4% PFA and stained with fuchsine. All samples were prepared in duplicates, of which cells and bacteria were counted in 6 representative details.

### RNA interference

For the knock-down of RhoGTPases, the predesigned and prevalidated siRNA oligonucleotides Hs RhoA-6, Hs Rac1-6 and Hs Cdc42-7 were purchased from Qiagen (Hilden, Germany). AllStars negative control siRNA (Qiagen) was used as a nonsilencing control. 3×10^4^ HeLa cells were transfected with siRNA (5 nM) in a 12-well plate 48 h prior to infection using HiPerFect transfection reagent (Qiagen) according to the manufacturer's fast forward protocol.

Successful knock-down was confirmed on RNA level by qPCR. Total mRNA of infected HeLa cells was extracted using RNeasy Mini Kit (Qiagen). 1 µg of total mRNA was reverse transcribed to cDNA using QuantiTect Reverse Transcription Kit (Qiagen). Real-time qPCR was performed on a LightCycler 480 (Roche Diagnostics, Mannheim, Germany) using QuantiTect SYBR Green PCR Kit and the respective QuantiTect Primer Assays (Rac1 1, RhoA 1, CDC42 2 and GAPDH 2). Relative expression levels were calculated using the ΔΔCT method [65]. Expression levels of the target genes were normalized to *glyceraldehyde-3-phosphate dehydrogenase* RNA expression. Thus, we could demonstrate at least 75% knock-down by the used siRNAs at mRNA expression level (data not shown).

### In vivo infections

C57BL/6 mice were purchased from Harlan Winkelmann (Borchen, Germany), TNF-Rp55^−/−^ [66] and IFN-γR^−/−^ [67] mice with a genetic C57BL/6 background were bred under specific pathogen-free conditions. Animal experiments were performed according to german law with permission of the Regierungspräsidium Tübingen. The experiments were performed with 6–8-week old female mice. To enable profound infection, 2.5 mg desferrioxamine (Sigma Chemical) in 200 µl PBS were administered to the mice intravenously one hour before infection. Mice were infected with 5×10^5^ bacteria from frozen stock suspensions in 200 µl PBS iv. into the tail vein. Uninfected control mice were in parallel treated with desferrioxamine. After two days, mice were sacrificed by CO_2_ asphyxiation and their spleens were surgically removed and placed in ice-cold HBSS (Ca^2+^ and Mg^2+^ free Hanks' balanced salt solution) (Biochrom) supplemented with 2% v/v fetal calf serum (FCS) (Sigma Chemical) and 10 mM HEPES buffer (Biochrom). Single cells were obtained by forcing the organs with a 5 ml syringe pestle through a 40 µm-pore nylon mesh cell strainer (Falcon; BD Labware, Franklin Lakes, USA). Cell suspensions were washed twice with ice-cold HBSS. Red blood cells were lysed from spleen samples by incubating the cell suspensions for 5 min at room temperature in lysis buffer (170 mM Tris, 160 mM NH4Cl, pH 7.4) followed by two washes in ice-cold HBSS.

### Detection of Bla-activity by microscopy

For the detection of Bla activity by immunofluorescence microscopy, HeLa cells were washed once with PBS and covered with 1× CCF4-AM staining solution supplemented with probenecid, prepared according to the manufacturer's instructions. Cells were incubated 30 min at a dark place at room temperature prior to observation with an Axiovert 200 microscope. Pictures were taken with an Axiocam HRc and Axiovision 4.4 Software was used to capture the shots and to produce overlay images (Carl Zeiss Microimaging, Esslingen, Germany). Filter sets for individual observation of coumarin and fluorescein fluorescence respectively were purchased from AHF (Tuebingen, Germany).

### Detection of Bla activity by flow cytometry

For the detection of Bla activity by flow cytometry, cells were resuspended in 1× CCF4-AM staining solution supplemented with probenecid, prepared according to the manufacturer's instructions (Invitrogen, Carlsbad, CA) and incubated 30 min at a dark place at room temperature prior to FACS analysis.

In case the detection of Bla activity was combined with the staining of leukocyte surface markers, 10^6^ cells per staining were resuspended in PBS. To avoid non-specific labeling, FcγII/III receptors were blocked by preincubation with mAb 2.4G2 for 20 min at RT.

Flow cytometry analysis was performed on a Dako Cyan cytometer using Summit 4.3 software (Dako, Carpinteria, CA) or on a BD Biosciences (Heidelberg, Germany) FACSCanto II using FACSDiva software. From each sample, at least 100,000 cells have been analyzed; error bars indicate the standard deviation between samples from different animals. An exemplary description of the steps how analysis was performed is shown in Figure S1.

### Immunohistology

For immunohistological analysis the tissues were embedded in Tissue-Tek OCT compound (Nunc, Roskilde, Denmark), snap-frozen in liquid nitrogen, and stored at −80°C. Frozen sections were prepared and stained by an immunoperoxidase method using 3,3-diaminobenzidine-tetrahydrochloride acid (DAB; Sigma, Deisenhofen, Germany) as chromogenic substrate. Nonspecific binding sites were blocked by incubation of the sections with PBS containing 10% fetal calf and 5% normal goat serum. For the yersiniae staining, rabbit anti-Hsp60 antibody was diluted 1∶200 in PBS containing 5% FCS and 5% normal goat serum for 1 h at room temperature. The secondary antibody was peroxidase-conjugated affinity purified F(ab')_2_ fragment goat anti rabbit IgG (Jackson ImmunoResearch; diluted 1∶200). Isotype-matched irrelevant rabbit IgG was used in controls and revealed no staining signal. The sections were counterstained with Mayer's hemalaun, mounted, and assessed microscopically on a BX51 microscope (Olympus Optical Co, Leinfelden, Germany) by two independent investigators. Pictures were taken with a DP71 camera and analysed with cell^B^ software (Olympus). Immunostaining of controls was negative for all groups tested.

### Statistics

If not otherwise stated, the means and standard deviations (SD) of data derived by cell culture experiments are calculated from four independent experiments. The number of mice which were used to calculate means and SD in mouse infection experiments is indicated in the manuscript or figure legends. Statistical analyses were performed using one-way ANOVA analyses with either Dunnett (comparison of control group with other groups) or Bonferroni corrections (comparison of all groups) as indicated in the figure legends or the text by using Graph Pad Prism software (GraphPad Software, La Jolla, USA). This software was also used to fit non-linear regression models.

## Supporting Information

Figure S1 Example for the flow cytometry analysis after in vivo infection. An example of the analyses for the determination of blue cells in splenocytes after E40-pOva and E40-pBla infection of mice is shown. (A) Forward and side scatter was analyzed of all dots measured. (B) High green fluorescence (green) characterizes uptake of CCF4 and therefore viability of cells, (C) shows as green dots that the green fluorescent viable cells are mostly found in R2. Gating with R1 and R2 results in the cell populations shown in D. (D) Cells in gate R3 were defined as blue cells.
(0.30 MB TIF)

Figure S2 Analysis of B cell subpopulations after in infection in vitro. Splenocytes were cultured and infected for one hour with or without E40-pBla (MOI 50). Cells were harvested and stained with anti-CD19-APC, anti-CD21-PE-Cy7 and anti-CD23-APC-Cy7 and then incubated with CCF4. Cells were analyzed by flow cytometry. Analysis was performed as followed. (A) Viable B cells were defined by gating using R1 & R2 & R3. All viable CD19^+^ cells (total) or blue CD19^+^ cells (R4) were analyzed for CD21 and CD23 expression. Percentages of CD21− (NFB, newly formed B cells), CD21^+^CD23^−^ (MZ, marginal zone B cells) and CD21^+^CD23^+^ (FO, follicular B cells) are indicated by numbers for this experiment. In total two experiments with similar results were performed. Mean and SEM of (B) the percentages of CD19^+^ cells, (C) the percentages of blue cells of the indicated B cell populations and (D) the percentages of the composition of total CD19^+^ blue cells are shown.
(0.45 MB TIF)

Figure S3 Analysis of activation of B cells in cell culture. Splenocytes were cultured and infected for one hour with or without E40-pBla. Cells were harvested and stained with anti-CD19-APC, anti-CD69-PE-Cy7 and MHC-II-APC-Cy7 and then incubated with CCF4. Cells were analyzed by flow cytometry. Analysis was performed in the following manner: (A) viable B cells were defined by gating using R1 & R2 & R3. (B) B cells were then further analysed for CD69 expression levels of all cells using additionally gate R5 (green+ blue+ cells), R6 (green+ blue− cells) or R7 (total). Histograms for CD69 expression of uninfected or E40-pBla infected cells are shown. Numbers indicate mean fluorescence intensities (MFI) for one representative experiment.
(0.33 MB TIF)

Figure S4 Infection of splenocytes of TNFR^−/−^, IFN-γR^−/−^ and C57BL/6 mice in cell culture. Splenocytes were infected with indicated MOI for one hour and subsequently cells were stained with CCF4-AM and analyzed by flow cytometry. Data summarize three independent experiments.
(0.07 MB TIF)
