# Supplementary figures and images for: Yersinia enterocolitica Targets Cells of the Innate and Adaptive Immune System by Injection of Yops in a Mouse Infection Model
Source: PLoS Pathog. 2009 Aug 14;5(8):e1000551. doi: 10.1371/journal.ppat.1000551 (PMC2718809; doi:10.1371/journal.ppat.1000551)

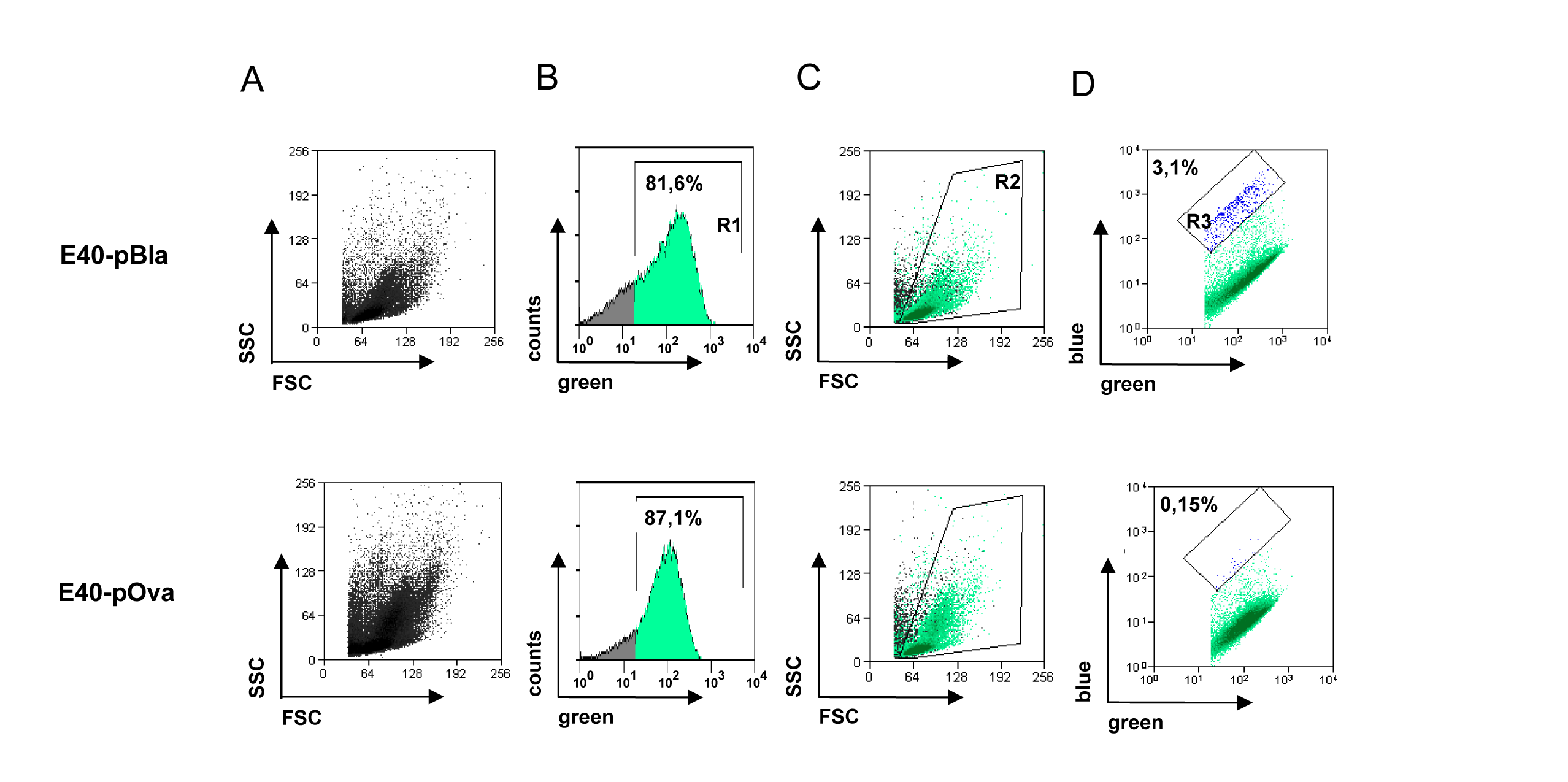

Supplement: Figure S1 — Example for the flow cytometry analysis after in vivo infection. An example of the analyses for the determination of blue cells in splenocytes after E40-pOva and E40-pBla infection of mice is shown. (A) Forward and side scatter was analyzed of all dots measured. (B) High green fluorescence (green) characterizes uptake of CCF4 and therefore viability of cells, (C) shows as green dots that the green fluorescent viable cells are mostly found in R2. Gating with R1 and R2 results in the cell populations shown in D. (D) Cells in gate R3 were defined as blue cells. (0.30 MB TIF) [file ppat.1000551.s001.tif]

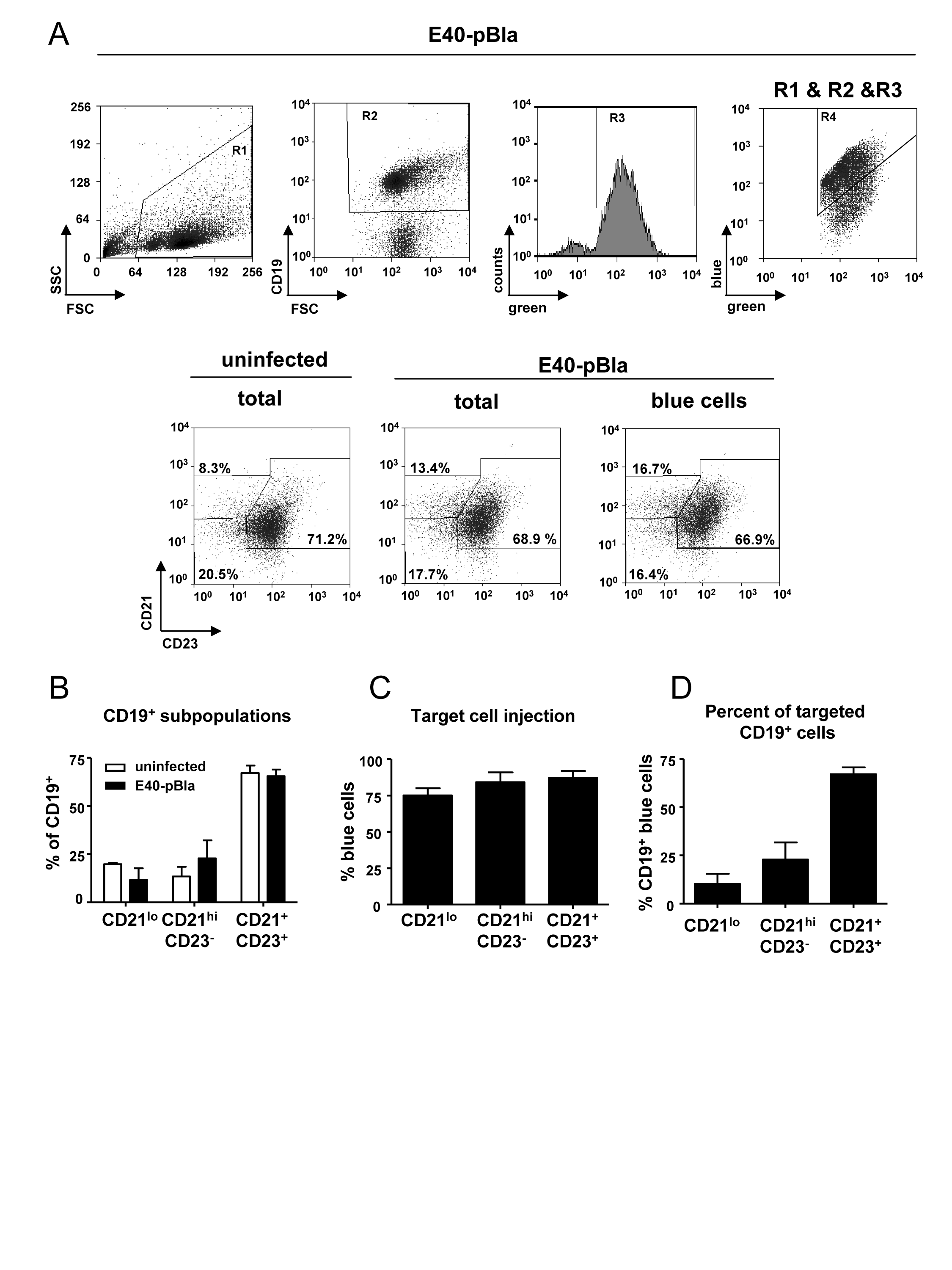

Supplement: Figure S2 — Analysis of B cell subpopulations after in infection in vitro. Splenocytes were cultured and infected for one hour with or without E40-pBla (MOI 50). Cells were harvested and stained with anti-CD19-APC, anti-CD21-PE-Cy7 and anti-CD23-APC-Cy7 and then incubated with CCF4. Cells were analyzed by flow cytometry. Analysis was performed as followed. (A) Viable B cells were defined by gating using R1 & R2 & R3. All viable CD19+ cells (total) or blue CD19+ cells (R4) were analyzed for CD21 and CD23 expression. Percentages of CD21− (NFB, newly formed B cells), CD21+CD23− (MZ, marginal zone B cells) and CD21+CD23+ (FO, follicular B cells) are indicated by numbers for this experiment. In total two experiments with similar results were performed. Mean and SEM of (B) the percentages of CD19+ cells, (C) the percentages of blue cells of the indicated B cell populations and (D) the percentages of the composition of total CD19+ blue cells are shown. (0.45 MB TIF) [file ppat.1000551.s002.tif]

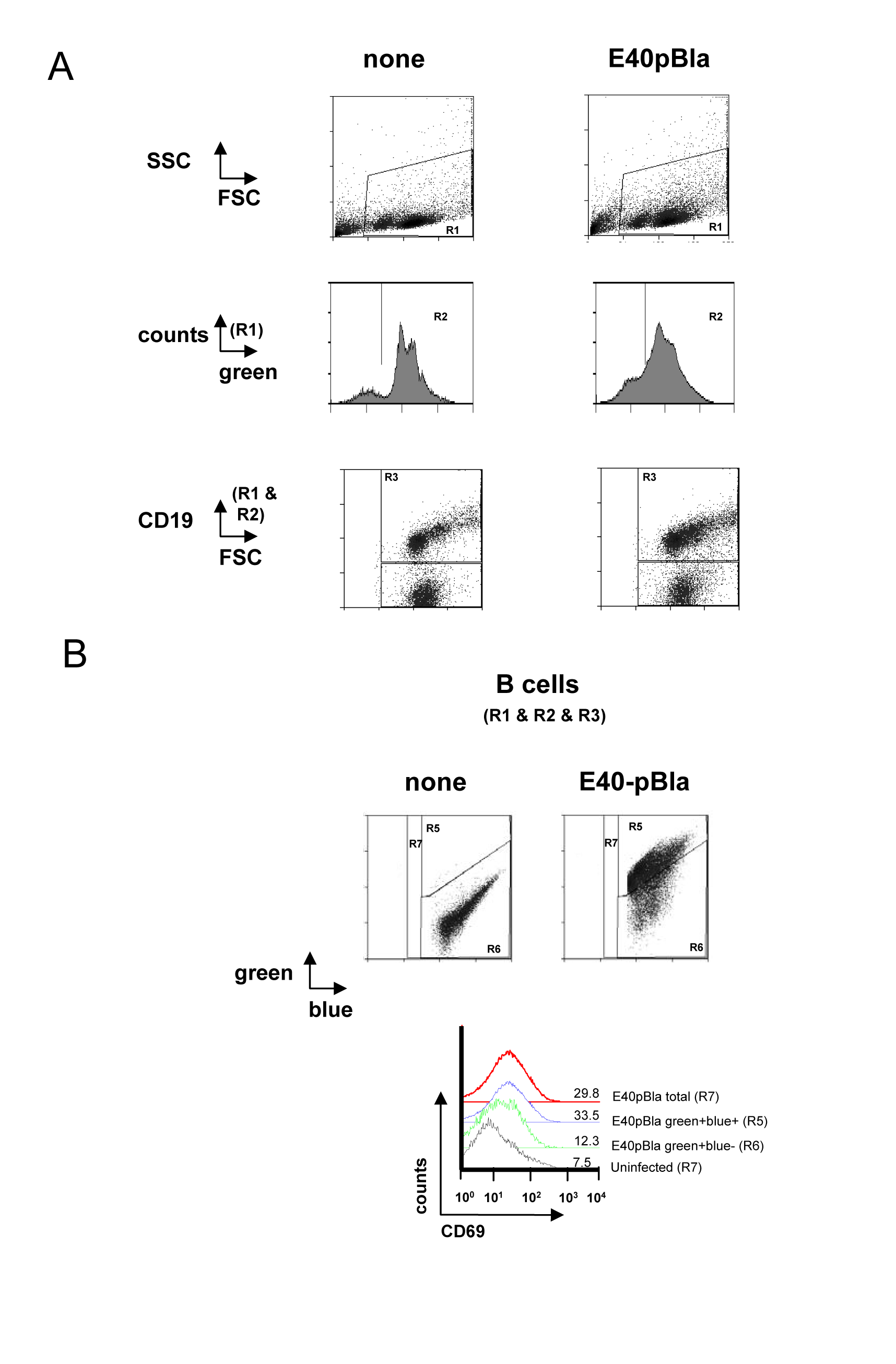

Supplement: Figure S3 — Analysis of activation of B cells in cell culture. Splenocytes were cultured and infected for one hour with or without E40-pBla. Cells were harvested and stained with anti-CD19-APC, anti-CD69-PE-Cy7 and MHC-II-APC-Cy7 and then incubated with CCF4. Cells were analyzed by flow cytometry. Analysis was performed in the following manner: (A) viable B cells were defined by gating using R1 & R2 & R3. (B) B cells were then further analysed for CD69 expression levels of all cells using additionally gate R5 (green+ blue+ cells), R6 (green+ blue− cells) or R7 (total). Histograms for CD69 expression of uninfected or E40-pBla infected cells are shown. Numbers indicate mean fluorescence intensities (MFI) for one representative experiment. (0.33 MB TIF) [file ppat.1000551.s003.tif]

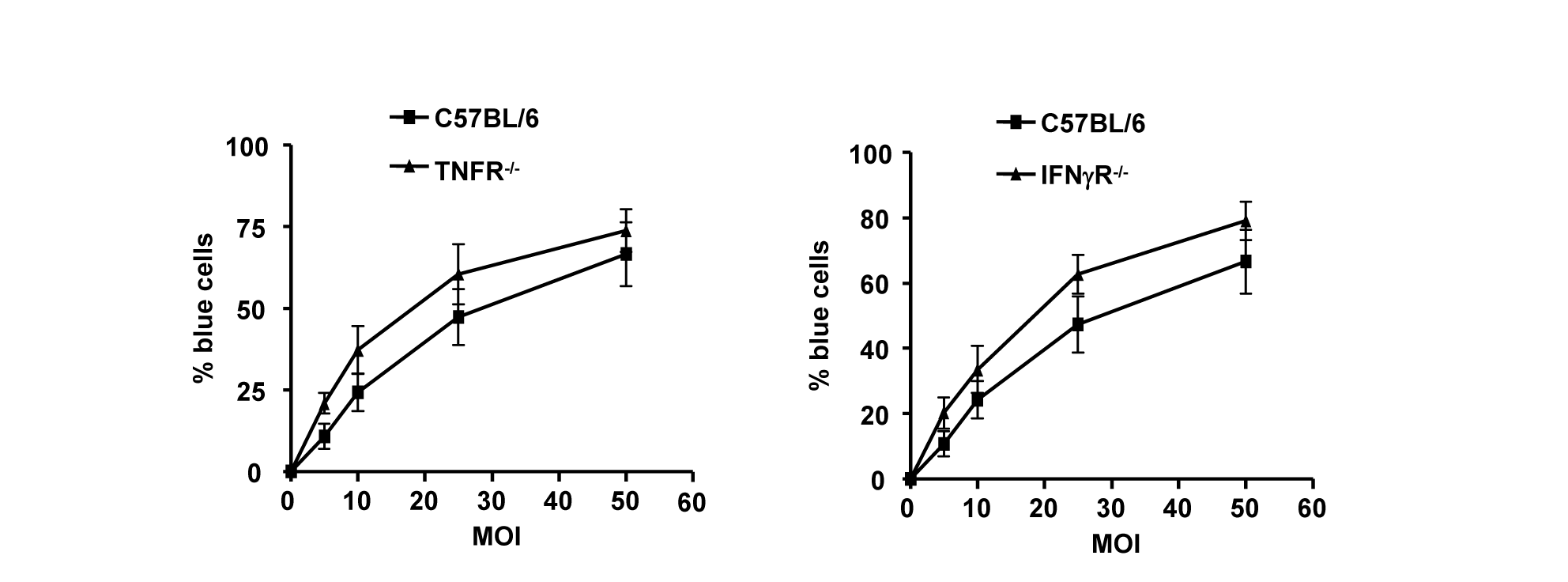

Supplement: Figure S4 — Infection of splenocytes of TNFR−/−, IFN-γR−/− and C57BL/6 mice in cell culture. Splenocytes were infected with indicated MOI for one hour and subsequently cells were stained with CCF4-AM and analyzed by flow cytometry. Data summarize three independent experiments. (0.07 MB TIF) [file ppat.1000551.s004.tif]
